# Future perspectives on novel CAR-T therapeutics beyond CD19 and BCMA in onco-hematology

**DOI:** 10.3389/fimmu.2025.1592377

**Published:** 2025-07-14

**Authors:** Alina Ershova, Alexandra Goldaeva, Alena Staliarova, Emil Bulatov, Alexey Petukhov, Nikolai Barlev

**Affiliations:** ^1^ Laboratory of Molecular Oncology, National Laboratory Astana, Astana, Kazakhstan; ^2^ Department of Biomedical Sciences, School of Medicine, Nazarbayev University, Astana, Kazakhstan; ^3^ International institute “Solution Chemistry of Advanced Materials and Technologies” (SCAMT), ITMO University, Saint Petersburg, Russia; ^4^ Dr. Sergey Berezin Medical Institute (MIBS), Saint Petersburg, Russia; ^5^ Institute of Fundamental Medicine and Biology, Kazan Federal University, Kazan, Russia; ^6^ Shemyakin-Ovchinnikov Institute of Bioorganic Chemistry, Russian Academy of Sciences, Moscow, Russia

**Keywords:** chimeric antigen receptor, CAR-T therapy, CAR-T target, hematological malignancies, oncology

## Abstract

CAR-T cell therapy is a type of adoptive immune therapy that relies on the specific targeting of cytotoxic T-cells to eliminate the malfunctioning cells in the body. Genetic engineering allows the generation of an almost infinite variety of chimeric antigen receptors (CAR) to ensure specificity for antigens on the surface of target cells. Therefore, CAR-T appears to be a powerful and versatile therapy for the treatment of various diseases, including cancer. Recently, CAR-T has emerged as a significant advancement in the management of hematological tumors, particularly B-cell malignancies, mainly due to the presence of specific antigens such as CD19 and BCMA. As a result, the market for CAR-T therapy is experiencing significant growth. However, the problem of relapses remains and warrants the search for new therapeutic approaches, including CAR-T technology. In this case, one of the major challenges is finding and evaluating new targets for CAR-T in terms of their likelihood of success. Here we propose a set of established criteria for the evaluation of potential targets for CAR-T cell therapy to treat hematological malignancies. These criteria include assessing the target in terms of its biological characteristics, such as expression level, cellular localization, tissue specificity, and clinical aspects, including unmet clinical needs and the success of clinical trials. Using these criteria, we validate our prediction of the next CAR-T cell therapy targets that will likely emerge soon.

## 1 Introduction

Understanding of the fundamental principles of the immune system’s physiology combined with the application of novel genetic engineering techniques have shifted the paradigm of cancer treatment towards the broader use of immunotherapy. The first evidence of cancer cure by the immune system was documented more than a century ago by an American surgeon William B. Coley, who was later called the “Father of Immunotherapy.” In his paper, he described cases of sarcoma regression in the context of acute bacterial infections, particularly erysipelas. He further hypothesized that infection-induced activation of the immune response could elicit an antitumor effect. These observations formed the conceptual basis for the subsequent development of cancer immunotherapy (1). Since then, immunotherapeutic cancer treatment methods have changed significantly and have become more effective. It is noteworthy that immunotherapeutic techniques were named «Breakthrough of the Year» in 2013 by the journal Science (2).

Today, immunotherapy covers a broad range of immunological methods, with cell-based immunotherapy as one of its subtypes. Chimeric antigen receptor (CAR) T-cell therapy is a pioneering approach in modern immuno-oncology that involves the adoptive transfer to a patient of their T-cells, which were genetically engineered to express chimeric antigen receptors specific for target tumor cells.

More than 30 years ago, Kuwana Y et al. (3) applied gene manipulation to create a chimeric antigen receptor (CAR) expression vector. Their work (1987) for the first time experimentally demonstrated that variable domains of antibodies could be functionally linked to constant regions of the T-cell receptor molecule. This became the first proof of the fundamental possibility of transferring an outstanding antibody specificity to T-cells whose T-cell receptors (TCRs) were not very discriminative. This discovery marked the beginning of the development of CAR-T therapy as a branch of immuno-oncology. Since then, numerous studies have been carried out to further develop the chimeric receptor design to make it more versatile and long-lasting yet specific. This approach quickly established itself as a promising, highly functional therapy for refractory and relapsed malignancies where other treatments failed to succeed. In 2014, the FDA granted *breakthrough therapy status* to Kymriah, a therapy consisting of CD19-directed CAR-T cells, the first CAR-T drug to be approved for clinical application (4).

The CAR-T cell therapy market is part of advanced medicine, one of the fastest-growing sectors of the pharmaceutical industry, which develops biological drugs. The market size was estimated at $2.6 billion in 2022 and is forecast to reach $35.9 billion by 2032, growing at a CAGR (compound annual growth rate) of 28.5% from 2023 to 2032 (5). In 2024, the website *bioinformat.com* published a summary table presenting 192 pharmaceutical companies that are developing various CAR-T cell therapy products (6).

Since 2017, several drugs have been developed for the therapy of hematologic diseases, such as B-cell acute lymphoblastic leukemia, large B-cell lymphoma, follicular lymphoma, mantle cell lymphoma (CD19-directed: Kymriah (7), Yescarta (8), Tecartus (9), Breyanzi (10), Carteyva (11), ARI-0001 (12), NexCAR19 (13), Obe-Cel (14)), and multiple myeloma (BCMA-directed: Abecma (15), Carvykti (16), Fucaso (17)). The resounding success of CAR-T in hematology is largely due to the availability of specific antigens, such as CD19 and BCMA, that are expressed on a very limited population of cells (18). It should be noted that CD19 is also expressed on healthy B-cells, leading to the phenomenon of B-cell aplasia after therapy. However, B-cell aplasia is considered as one of the expected pharmacodynamic effects of CD19-targeted CAR-T therapy that destroy B-cells. This condition is usually well controlled by regular immunoglobulin replacement therapy. B-cell repopulation depends on the duration of CAR-T cell persistence but usually completes after a year. Thus, temporary loss of normal B-cells is considered as an acceptable and clinically manageable consequence of the effective therapy (19, 20).

Despite significant advances in the treatment of hematological diseases, CAR-T cell therapy still faces various challenges, including disease relapse and refractory cases. For example, acute lymphoblastic leukemia (ALL) has a 30-60% relapse rate after therapy (21). Additionally, antigen-negative relapses frequently occur (21), requiring a reconsideration of treatment strategies. Therefore, there is a continuing need to develop new, optimized approaches for targeting various hematological diseases with CAR-T cells.

Ideally, an antigen that is chosen to be targeted by CAR-T should be present only on the surface of tumor cells. However, this condition is almost impossible to achieve *in vivo* because most of the surface antigens are shared between the tumor cells and normal cells. Therefore, in this work we aimed to define the criteria that are critical for the target antigen selection to be used in CAR-T therapy. We considered the following parameters: expression level, localization, tissue specificity, unmet clinical need, and success in clinical trials. Accordingly, we reviewed the list of CAR-T targets currently being explored in clinical trials and, using the criteria we have identified, attempted to predict the next approved targets for future CAR-T products in onco-hematology.

## 2 General principles of CAR design and domain architecture

**Figure 1:**
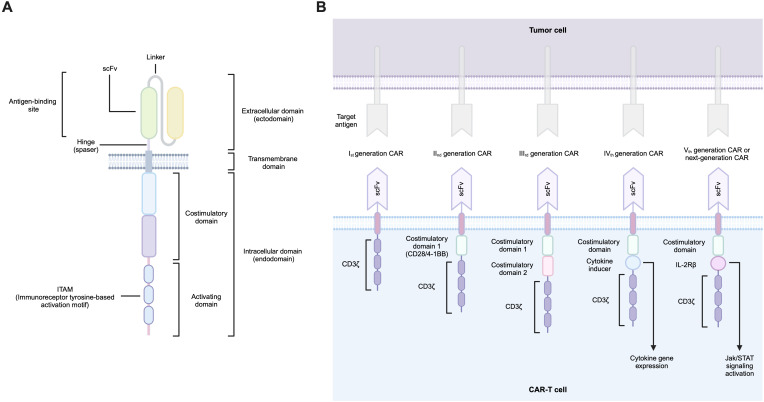
**(A)** CAR structural elements, **(B)** Evolution of CAR structure.

The CAR design is similar for both hematological and solid tumors and is based on a modular organization. A typical CAR consists of several functional domains, including an antigen recognition domain, a hinge region (spacer), a transmembrane domain, and finally, an endodomain that transduces the activation signal from the outer membrane receptor to the nucleus (**Figure 1A**).

The original design of the CAR antigen-binding domain was based on a single-chain variable fragment (scFv) of a monoclonal antibody that selectively recognizes the antigen. This minimal fragment consists of the immunoglobulin heavy chain (VH) and immunoglobulin light chain (VL) variable domains joined by a flexible linker. The reason for using antibody fragments in the design of CARs stems from the fact that immunoglobulins have the highest specificity towards the corresponding antigen. All CAR-T products approved to date contain such an scFv-based extracellular domain, with the only exception of Carvykti (ciltacabtagene autoleucel; Janssen Biotech, Inc.), which is based on two single-domain antibody fragments called nanobodies that bind to two BCMAs epitopes (22–26). These unconventional antibodies found in certain members of the Camelidae family contain only the heavy chain (VHH), which lacks the CH1 domain required for the light chain pairing. Nevertheless, their binding affinity for the target antigen remains intact (27).

In addition to scFv and nanobodies, variable new antigen receptors (vNARs) are viable alternatives to the immunoglobulin-based antigen-binding domains. The vNAR is a variable fragment of a new antigen receptor (IgNAR) antibody isolated from sharks that also contains only heavy chains (28). It has been shown that vNAR-based CAR-T cells could be effective candidates for therapy for PDL1-positive solid tumors (29). Although not yet used in treatment of hematological diseases, they serve as an example of structural diversity in the field of CAR receptor design.

In theory, any molecule with an affinity for an antigen of interest can function as an antigen-binding domain. In this respect, ligand-based CARs were shown to be promising new tools: the FLT3L or GM-CSF-specific ligands were proposed as antigen-binding domains for CARs that target the FLT3-positive acute myeloid leukemia (AML) (30, 31). The BAFF ligand CAR-T cells were generated to target the BAFF receptors, which are expressed on B-cell malignancies (32). One of the best-known receptor-based CAR variants is the CAR based on the NKG2D receptor that was shown to be effective against tumor cells expressing the NKG2D ligands (33). Furthermore, certain cytokines could also be a valuable source for antigen-binding domains: zetakine CARs were designed to target the decoy IL-13 receptor α2 (IL-13Rα2), which is expressed in 60% of glioblastoma tumors. Such a CAR contains mutant IL-13 (E13Y) as an antigen recognition region (34).

Furthermore, non-immunoglobulin engineered binding scaffolds that can recognize and bind a target molecule can also be employed as CARs (35). For instance, it has been demonstrated that DARPin-based CAR-T cells (designed ankyrin repeat proteins) can offer a compelling alternative to the conventional scFv CAR-T cells (36). Importantly, DARPin-based CAR-T has the potential to target multiple antigens simultaneously, a feature that can prove advantageous in case of antigen loss in cancer cells (37).

The hinge provides ample mobility to the antigen-binding domain, facilitating its optimal binding to the desired antigen site. It is important to note that the activation of T-cells by the CAR-antigen target interaction depends largely on the steric conditions of the antigen site, its proximity to the target cell membrane, and its accessibility. Such nuances may be important for the optimal CAR design and, in particular, for choosing the length of the spacer region of the molecule to achieve optimal CAR-T cell signaling (38). The spacer regions for CARs mainly derive from two groups of molecules: Ig-based hinges (e.g., IgG1, IgG2, IgG4) and non-Ig-based hinges from CD28 and CD8 (39).

The transmembrane domain is located between the spacer and the endodomain and is used to anchor the chimeric receptor to the cell membrane. This domain is most commonly derived from fragments of CD4, CD8, CD28, and CD3ζ molecules (40).

The endodomain of CAR is responsible for the transmission of a signal from the ectodomain to the T-cell transcriptional machinery. The endodomain comprises a costimulatory domain and an activation domain. A wide range of possible molecules are utilized as its backbone. It is the co-stimulatory CAR domain that carries the most diversity. The current FDA-approved CAR-T products contain CD28 and 4-1BB as costimulatory domains and CD3ζ as an activating domain (41). The prevalent costimulatory domains for CARs are derived from two major superfamilies of T-cell co-receptors: IgSF (e.g., CD28, ICOS) and TNFRSF (e.g., 4-1BB, OX40, and CD27). Furthermore, costimulatory domains based on molecules such as IL-15R-α, IL-2Rβ, MyD88, and CD40 may also be utilized (42). CD3ζ carries three ITAM motifs that mediate activation signaling conduction. CAR-T technology traditionally used CD3ζ as the activation molecule. Recently, it has been demonstrated that incorporating alternative TCR chains (CD3δ, CD3ϵ, CD3γ) may provide opportunities to enhance CAR-T cell technology and modulate its activity (43).

CAR technology extends beyond the receptor structure described herein (**Figure 1B**), encompassing diverse cell populations as CAR-carrying cells, as well as numerous genetic modifications to both receptors and cells themselves. Despite the wide repertoire of CAR design possibilities and the diversity of modifiable cell populations, the choice of antigen for CAR targeting remains a paramount challenge.

## 3 Criteria for CAR-T antigen selection

Currently, there are no universally accepted criteria for the evaluation and validation of potential targets for CAR-T therapy. The criteria proposed in this manuscript apply to both solid and hematological neoplasms and represent a systematic approach to the selection of therapeutically relevant targets. In general, a molecule is considered an ideal target for CAR-T if it demonstrates stable expression among the population of malignant cells with no detectable expression of this antigen among normal cells. However, as mentioned previously, there are very few molecules that fulfill such parameters. In ongoing clinical trials, antigens that are targeted by CAR-T therapy are expressed in both normal and malignant tissues (44). A promising target should therefore meet a new criterion–an improved risk-benefit ratio (45). However, the risk-benefit ratio is influenced by multiple factors which are often difficult to calculate. It depends on both specific therapy and clinical situation. In general, benefits include high response rate, durable remission, and long-term survival, whereas risks comprise CRS, neurotoxicity, secondary T-cell malignancies and hospital infections. Factors such as patient preferences, provider biases, and economic considerations all play roles in this multifactorial event, making it difficult to quantify. Thus, the perception of the risk-benefit ratio can vary significantly based on characteristics of an individual patient, physician viewpoints, and systemic factors. This complexity increases even further when dealing with pain treatment, as the subjective nature of pain and the lack of data on the intervention outcomes complicate the decision-making process (46).

Given that no clear criteria have been defined, several studies have been devoted to finding criteria that at least approximate the potential target.

In 2009, a pilot project proposing a system for the distribution and evaluation of cancer vaccine target antigens was launched by the National Cancer Institute (NCI). Using analytic hierarchy process (AHP), the priority of various criteria was ranked in descending order, as follows: 1) therapeutic function, 2) immunogenicity, 3) role of the antigen in oncogenicity, 4) specificity, 5) the expression level and percentage of antigen-positive cells, 6) stem cell expression, 7) number of patients with antigen-positive cancers, 8) number of antigenic epitopes, and 9) cellular location of antigen expression (47). In 2023, M. M. Jin and E. von Hofe mentioned this article and emphasized that some of the criteria can be reliably used to evaluate the target for CAR-T, as currently most clinical trials against solid tumors are being conducted against Tumor Associated Antigens (TAAs), which were prioritized in the 2009 study (48). Currently, the influence of the immunosuppressive microenvironment should also be considered when selecting parameters for targeting CARs, as it significantly affects the persistence and efficacy of CAR-T cells (49).

In 2019, Wei, J., Han, X., Bo, J., et al. highlighted the three most important parameters in evaluating a CAR-T target: coverage and specificity, assuming that the molecule should have sufficient coverage over the tumor cells and be expressed selectively on a subsequent cell population to avoid severe toxicities (50). In addition, stability is the third critical factor in evaluating a potential molecule because the more unstable the target, the easier it is for cancer cells to escape.

A potential target for CAR-T should demonstrate efficacy and safety in preclinical studies, such as *in vitro* assays and/or animal models. To have potential as a therapeutic target, the preclinical efficacy score should also be considered as a parameter when evaluating the target (51). In addition, a panel of tests is required to assess the CAR effector function, including cytokine production assays, flow cytometric analyses to assess killing potency (by counting remaining viable target cells), and CAR-T cell proliferation and phenotype (51), which can be achieved by TAA-dependent cytotoxicity assessment and tumor-derived organoids for CAR-T cell therapy (52).

*In vivo* models will allow the study of CAR-T cell behavior in real-life settings, including 1) CAR-T function in established large tumors, 2) the dynamics of the antitumor effect during treatment, 3) the crosstalk between CAR-T cells and the host immune system, 4) the trafficking of systemically injected cells into solid tumors, and 5) therapy-related toxicity (51).

Engineered CAR-T cells should be monitored for «on-target off-tumor» and off-target effects due to the high expression of their targets in different tissues and cell types, as well as potential cross-reactivity with proteins closely associated with the target molecule (45). In addition, the CAR-T cell infusion itself should be evaluated before approval for clinical trials: sterility, cell viability, purity (cleanliness of bead removal), identity (percentage of T-cells), and CAR expression and potency (51).

To ensure product safety before clinical trials, the FDA released “Considerations for the development of Chimeric Antigen Receptor (CAR) T-cell products” in 2022 (53).

**Figure 2:**
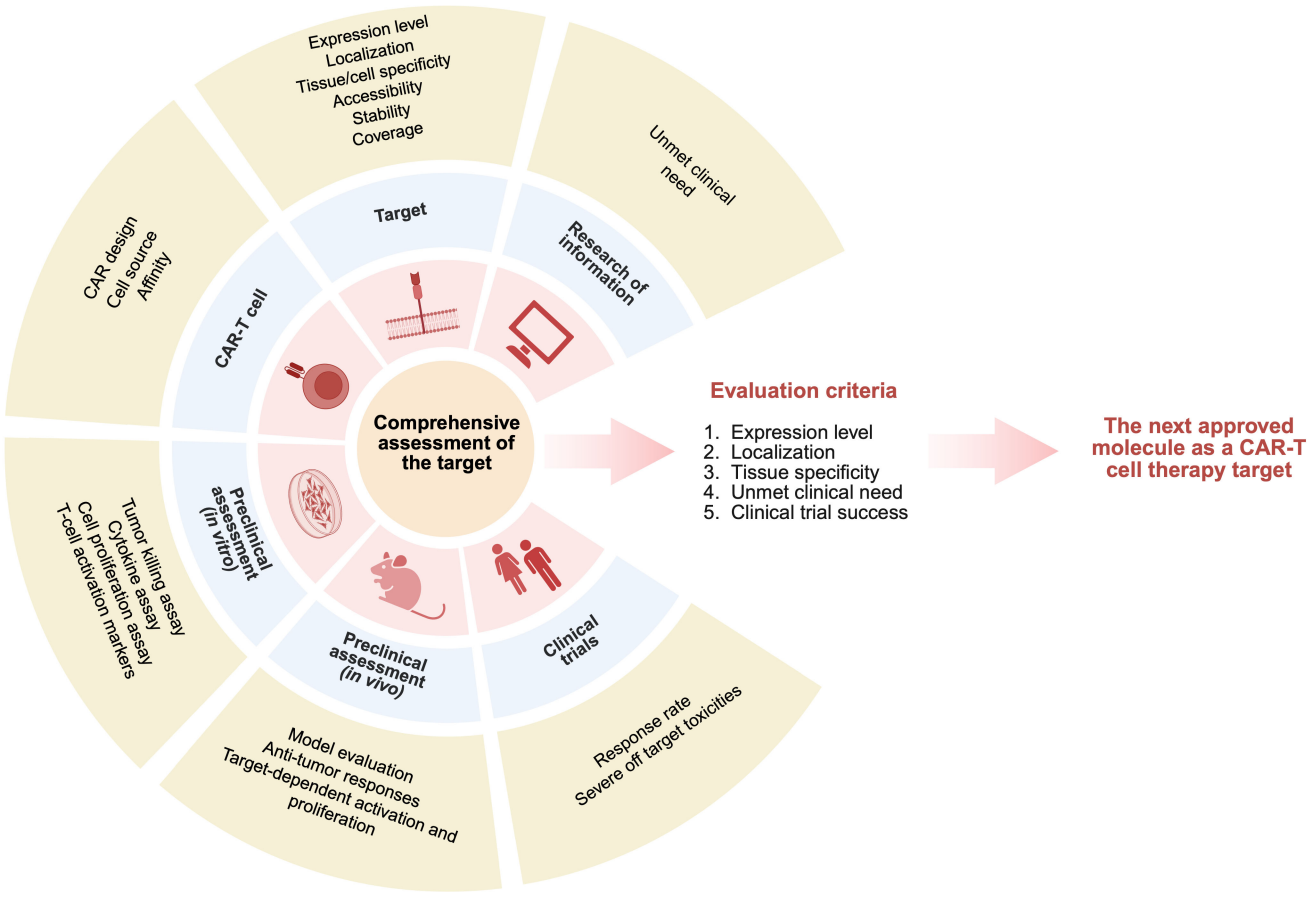
Comprehensive criteria for evaluating potential targets across all stages of CAR‑T development.

As we are collecting the data on clinical trials, we do not consider the sub-criteria of *in vitro* and *in vivo* studies separately, unless the target is in clinical trials and it has already been validated *in vivo* and *in vitro (* 51). We identified the main criteria that can be evaluated using the open-source databases and articles: 1) expression level, 2) localization, 3) tissue specificity, 4) unmet clinical need, and 5) clinical trial success (**Figure 2**).

Based on the biological data, targets can be ranked according to: 1) target expression (the target should have a restricted expression area to minimize «on-target off-tumor» effect and reduce toxicity), 2) tissue specificity (the target should be specific for cancer cells), and 3) localization (to ensure surface expression to be biologically available for CAR-T targeting).

Based on the characteristics of a particular tumor, we assess: 1) disease prevalence, 2) progression-free survival on first-line therapy, 3) event-free survival in case of disease relapse, and 4) number of lines of therapy used before CAR-T therapy. Thus, by summarizing information about the disease (patient outcomes, survival rates), we can estimate criteria such as unmet clinical need and provide information on whether this clinical need can be addressed by using CAR-T therapy. To validate the success of an investigational CAR-T therapy, we propose to assess clinical success (response rate during CAR-T therapy) and safety (a target should not pose a significant safety risk to patients, i.e., it should not be associated with serious adverse events or toxicities).

## 4 Evaluation of selected targets in onco-hematology based on the proposed criteria

To assess potential niches and targets for the development of a new CAR-T therapy, we referred to biotechnological companies’ pipelines. We identified 24 CAR-T target variants in onco-hematology, including approved targets, dual and multiple targeting options, targets approved for other therapies, and novel agents (Supplementary Information **“** **Supplementary Table S1**”). Next, based on suggested criteria, we evaluated the selected targets (CD19, BAFFR, BCMA, CD22, CD20, CD123, CD33, CD38, CD70, CLL1, CD30, GPRC5D, CS1, ROR1, TRBC1, CD7, CD5).

### 4.1 Biological characterization

In order to characterize the candidate genes, we briefly analyzed the available information from public sources such as the HPA, GTEx, and UniProt databases (see **Supplementary Information** **“**Methodology” for a detailed description of the analyses we performed).

The study considered the cellular localization of molecules, the tau parameter of specificity of gene expression (tau specificity score, τ) in tissues (54), as well as RNA and protein expression in various normal tissues and RNA expression in immune cells, as the focus of this paper is on onco-hematological diseases. Our analysis suggests that the optimal candidate gene should meet the following parameters: RNA and protein expression should be maximal and highly restricted in the immune system (ideally absent in other tissues), and the RNA expression in immune system cells should be restricted to one cell type, if possible.

**Figure 3:**
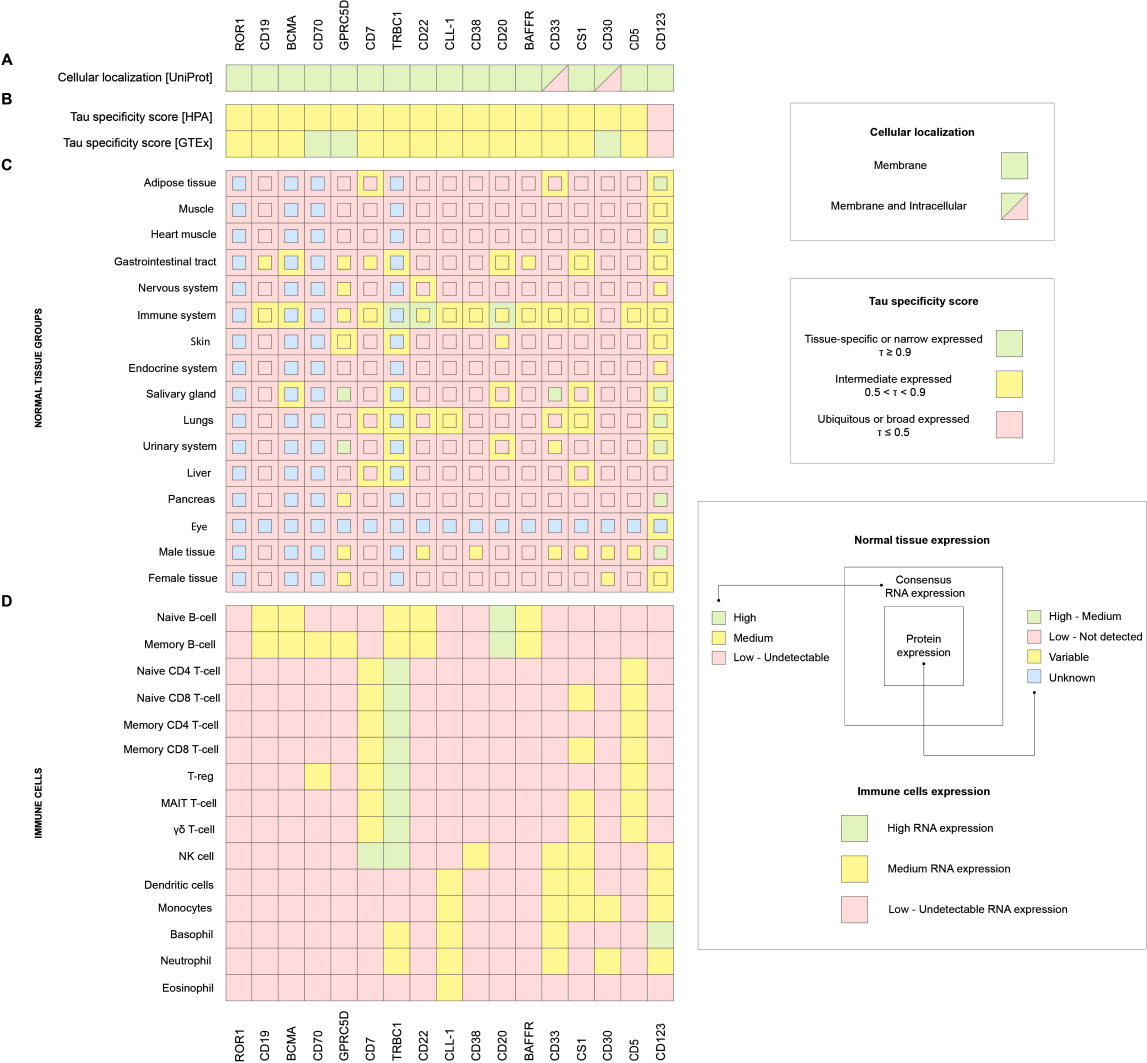
**(A)** UniProt cellular localization, **(B)** HPA and GTEx Tau (τ) specificity score of gene expression in normal tissues, **(C)** HPA consensus RNA expression in normal tissues (external square), HPA protein expression in normal tissues (internal square), **(D)** HPA RNA expression in immune cells.

Thus, the obtained results were analyzed and presented in **Figure 3**. One of the key parameters, τ, reflecting the tissue specificity of expression, showed that most of the genes under consideration have moderate (intermediate) specificity, that is, they are expressed predominantly in certain cell types (**Figure 3B**). However, upon closer examination, it becomes apparent that each gene has its unique expression profile, highlighting the need for an individual approach when evaluating them in the context of CAR-T therapy. Analysis of the subcellular localization of the target candidate molecules showed that most of them are membrane-bound, which is essential for the successful recognition and activation of CAR-T cells. The exceptions are CD33 and CD30, for which localization can vary from the cell membrane to intracellular depending on the isoform, (**Figure 3A**).

According to the databases, ROR1 expression appears to be very low or undetectable in all tissues and cell populations examined (**Figures 3C, D**). A similar pattern is observed for CD70, except for moderate expression in two immune cell types, B cells and T regulatory cells (Tregs) (**Figures 3D**). In the case of GPRC5D and CD123, strong expression is observed in several non-target tissues, including the gastrointestinal tract, nervous system, salivary glands, and urinary system, etc., which significantly limits their applicability as selective targets for CAR-T therapy (**Figure 3C**). There are no protein expression data for the TRBC1 target, but the transcriptomic analysis indicates the presence of mRNA in tissues outside the immune system, such as the lung, liver, and gastrointestinal tract. This may be due to the infiltration of these organs by T cells, since TRBC1 encodes the β-chain of the T-cell receptor (TCR β constant region 1). Moreover, TRBC1 expression is detected not only in T cells, but also in B-cells, NK cells, basophils, and neutrophils, which calls into question its strict restriction to a single cell type (**Figures 3C, D**). CLL-1 demonstrates expression in various immune cell populations, including dendritic cells, monocytes, basophils, neutrophils, and eosinophils (**Figure 3D**), potentially increasing the risk of off-target effects. Finally, CS1 is characterized by broad expression throughout the immune system, including both innate and adaptive components (**Figures 3C, D**).

CD19, BCMA, CD20, CD22, CD38, BAFFR, CD7, and CD5 were identified as the most appropriate target genes. CD19 and BCMA are established target genes for CAR-T therapy, based on the list of currently approved drugs (although information on protein expression for BCMA is limited in our analysis). RNA and protein expression analyses predominantly concentrate on B-cells (**Figure 3D**). CD20, CD22, and BAFFR are the markers for identifying B-cell populations due to their protein expression being almost exclusive to B-cells (**Figure 3D**). CD38 shows significantly high expression of both RNA and protein in the immune system (**Figures 3C, D**). CD5 and CD7 are exclusively located in the T-cell compartment of the immune system and exhibit an almost entirely specific pattern of expression (**Figures 3C, D**).

### 4.2 Assessment of unmet clinical needs in onco-hematologic disorders

#### 4.2.1 Acute lymphoblastic leukemia

In 2022, the NCI’s Surveillance, Epidemiology, and End Results (SEER) database found that ALL incidence peaked at 4.2 per 100,000 in patients under 15 and 1.8 per 100,000 for patients over 65. The incidence rates in the 15–64 age group, which covers the able-bodied population, were 1.2 and 1.0 per 100,000 population for 15–39 and 40–64 years, respectively.

The 5-year relative survival rate for children and adolescents under the age of 20 is 92% with first-line therapy. From 2015 to 2021, the 5-year relative survival rate for ALL patients of all ages in the US was 72.6%. The event-free survival rates for second-line therapy differ significantly between adult and pediatric patients. The multicenter ALL-REZ-BFM 2002 and COG AALL1331 studies showed the highest survival rates for relapsed ALL in children. In ALL-REZ-BFM 2002 (538 relapsed ALL patients, 420 randomized), 5-year overall and event-free survival rates were 69% and 60%, respectively. In COG AALL1331, of 220 relapse ALL patients, 208 underwent randomization. The bispecific antibody randomization branch had the highest 2-year overall and event-free survival rates – 79.4% and 59.3%, respectively (54, 55).

Even though 80-90% of adult patients react to induction chemotherapy, only 30-40% attain long-term remission (56). Children have higher survival rates than adults for relapsed ALL; the 3- and 5-year event-free survival (EFS) for second-line therapy are 24% and 10%, respectively (57). The findings of ALL relapse therapy suggest finding new, more effective therapies.

#### 4.2.2 Acute myeloid leukemia

AML is the most common form of acute leukemia in adults, accounting for about 80% of all cases (58), with the incidence increasing with age (59). According to the SEER database, the peak incidence of AML in the US in 2022 was 21.2 per 100,000 standard US population for patients over 65 years old, 3.8 per 100,000 for 40-64-year-olds, and 0.7 and 1.3 per 100,000 for those under 15 years old and 15–39 years old, respectively. Long-term survival in this condition depends on patient age, leukemic cell cytogenetic and molecular genetic alterations, and therapeutic responsiveness. AML treatment is complicated because intensive chemotherapy regimens, including high doses of cytarabine in combination with anthracyclines and allogeneic transplantation of hematopoietic stem cells for high-risk patients, are needed to achieve complete remission and reduce the risk of disease relapse (60). Despite treatment, AML survival rates are low. From 2015 to 2021, the 5-year relative survival rate for AML patients of all ages in the US was 32.9%, and for those under 20, it was 71,5% (62). 5-year overall survival rates for adult patients with relapsed acute myeloid leukemia do not exceed 19% (63).

The Berlin-Frankfurt-Munster Study Group (BFM) and the Children’s Oncology Group (COG) showed therapeutic results in pediatric patients with first-relapse AML in 2021. The BFM and COG investigations enrolled 197 and 852 participants. The 5-year overall survival rate of relapsed AML patients under BFM group therapy was 42%, and COG was 35% (64).

Combinations of chemotherapeutic medicines with targeted agents and monoclonal antibodies are being studied for AML treatment. Since 2017, 10 drugs have been approved by the FDA to treat AML: FLT3 mutation inhibitors (gilteritinib, midostaurin, quizartinib (Vanflyta)), BCL-2 inhibitors (venetoclax), IDH1 and IDH2 inhibitors (ivosidenib, enasidenib), CD33-directed monoclonal antibodies and antibody-drug conjugates (ADCs) (gemtuzumab ozogamicin), liposomal cytarabine and daunorubicin in a fixed molar ratio of 5:1 (CPX-351), Hedgehog signaling pathway inhibitors (glasdegib), oral hypomethylating agent azacitidine (CC-486), and oral hypomethylating agent decitabine-cedazuridine (65, 66). MD Anderson reported that even with modern combined therapy regimens, adult patients had a 5-year survival rate of 40-50%, while senior patients (over 60) had not exceeded 30% (61). Thus, morbidity and survival rates, especially in older AML patients, suggest the need for more effective and less harmful treatments for AML patients.

#### 4.2.3 Multiple myeloma

MM, one of the most frequent malignant hematological illnesses, rarely occurs in children and adolescents, but it increases with age (67). According to SEER database in 2022, the highest incidence of MM was 40.3 per 100,000 US population at 65 years old, 8.4 at 40–64 years old, and 0.3 at 15–39 years old.

Over 15 new medications and 33 novel MM treatment regimens have been licensed in the past two decades. Current disease treatment strategies use proteasome inhibitors, immunomodulatory medicines, and a monoclonal antibody to CD38 (daratumomab). For newly diagnosed and relapsed and refractory (r/r) MM, triple and quadruplet therapy using these types of medicines and dexamethasone are conventional treatments (68). New medications and therapies have improved MM patients’ survival rates. For instance, the 5-year relative survival rate has grown from 34.5% in 2000 to approximately 60% now (68).

Thus, SEER data showed a 62.4% 5-year relative survival rate for US MM patients of all ages with first-line therapy from 2015 to 2021. Tumor cell resistance causes disease relapse, making MM treatment difficult despite advancements.

Immunomodulatory drugs, proteasome inhibitors, and monoclonal antibodies in the initial phase of therapy and the first relapse have necessitated more intensive combination therapy in patients with second or more relapses, including antibody-drug conjugates, bispecific T-cell activators, and T-cells with chimeric antigen receptors.

#### 4.2.4 Non-Hodgkin’s lymphomas

NHL are the most frequent malignant hematological diseases worldwide (69). According to SEER, the 2022 US incidence rates for 15-39-year-olds, 40-64-year-olds, and 65-year-olds are 4.2, 19.7, and 87.7 per 100,000 population (70). Thus, NHL incidence increases with population age, particularly in middle-aged (40–64 years) and elderly individuals. Novel therapeutic drugs such as anti-CD20 antibodies, BCL-2 inhibitors, and PD-1 inhibitors have improved disease treatment results (71). NHL has a 74.2% relative survival rate in the US from 2015 to 2021. Children and young people under 20 with NHL had higher 5-year relative survival rates at 91.3%.

Relapses are NHL therapy’s biggest issue. First-line NHL treatment has promising results, but relapse treatment in all NHL forms is inadequate. Ayers EC, Li S, Medeiros LJ, et al. found that adult patients with relapsed aggressive B-cell malignant lymphoma (diffuse LBCL, B-cell lymphoma of high malignancy) had 24% overall survival and 14% 3-year progression-free survival (72). The timing of relapse/progression determined r/r follicular lymphoma (FL) survival. FL progression/relapse within 24 months of chemotherapeutic treatment reduced 5-year survival to 34-50%. The 5-year survival rate of late relapse/progression patients (greater than 24 months after chemotherapy) was 90% (73, 74).

The International Berlin-Frankfurt-Münster Group (I-BFM) and the European Child and Adolescent Non-Hodgkin’s Lymphoma Group (EICNHL) studied 639 pediatric patients with relapsed NHL of various types. Relapsed Burkitt’s lymphoma had a 28% 8-year overall survival rate, diffuse 50%, primary mediastinal B cell 57%, T-lymphoblastic 27%, and B-lymphocyte precursor 52% (75). Thus, relapsed NHL treatment results show the need for new, effective treatments (76).

#### 4.2.5 Hodgkin’s lymphoma

According to the SEER database, the high incidence rate of HL in 2022 was in two age groups: 15–39 years old and 65 years of age and older were 3.5 and 3.6 per 100,000 population, respectively. The lowest incidence rate was in the age group under 15 years of age, at 0.5 per 100,000 in the United States, and in the 40–64-year-old group, at 2.3 per 100,000 in the US. The use of modern risk-adapted first-line chemotherapy in HL patients has resulted in high survival rates. According to the SEER database in the United States, in the period from 2015 to 2021, the five-year relative survival rates of patients with HL of all age groups, as well as the analyzed value in the age group under 20 years, were 89% and 98.9%, respectively. The first-line intensive combination of chemotherapy schemes and radiation therapy has resulted in 10-year overall and event-free survival rates exceeding 90% and 80%, respectively. In turn, when used in patients with advanced stages, 10-year event-free survival rates of more than 80% were achieved (77). After first-line therapy, approximately 10% of patients with early-stage HL and 20–30% of those with advanced-stage HL experience either primary refractory disease or relapse, necessitating further treatment to achieve long-term remission (78). Currently, new biological treatments are being used in several studies alongside standard therapy, including the anti-CD30 antibody drug conjugate (brentuximab vedotin) as well as antibodies to PD1 (nestulumab, pemprolizumab), which aim to minimize the toxicity of therapy while maintaining high efficacy.

#### 4.2.6 Summary

Based on the records of malignant hematological diseases in the United States in 2022, it seems reasonable to assume that the most frequently diagnosed diseases among the working population of the United States are non-Hodgkin’s lymphomas and multiple myeloma (**Table 1**). The main problem in the treatment of these diseases is relapses, since survival rates for relapsed lymphoma and MM are significantly lower than the results of primary chemotherapy treatment. In this regard, new approaches and methods for treating these pathologies are required. Among them, the most promising and clinically proven is CAR-T cell therapy.

**Table 1:** Age‑adjusted incidence rates and outcomes in patients with onco-hematological diseases.

Metrics	Acute lymphoblastic leukemia (ALL)	Acute myeloid leukemia (AML)	Multiple myeloma (MM)	Non-Hodgkin’s lymphomas (NHL)	Hodgkin’s lymphoma (HL)
Age‑adjusted incidence rates per 100,000 (2022, SEER): patients of all ages	1.9	4.5	7.8	18.8	2.5
Age-adjusted incidence ratesper 100.000, 2022 year (SEER): patients under 15 years of age	4.2	0.7	0	1.2	0.5
Age-adjusted incidence rates per 100.000, 2022 year (SEER): patients aged 15–39 years	1.2	1.3	0.3	4.2	3.5
Age-adjusted incidence rates per 100.000, 2022 year (SEER): patients aged 40–64 years	1.0	3.8	8.4	19.7	2.3
Age-adjusted incidence rates per 100.000, 2022 year (SEER): patients aged 65 years or elder	1.8	21.2	40.3	87.7	3.6
5‑year relative survival rates (2015 – 2021): patients under 20 years of age	92%	71.5%	–	91.3%	98.9%
5-year relative survival rates (2015 – 2021), patients of all ages	72.6%	32.9%	62.4%	74.2%	89%
Relative survival rates in case of disease relapse: patients under 20 years of age	ALL-REZ-BFM 2002: 5-year OS – 69%; 5-year EFS – 60.0%. COG AALL1331: 2-year OS – 79.4%; 2-year EFS – 59.3% (blinatumomab)	BFM: 5-year OS – 42%; COG: 5-year OS – 35%		Burkitt lymphoma/leukemia: 8-year OS – 28%; Diffuse large B-cell lymphomas: 8-year OS – 50%; Primary mediastinal large B-cell lymphomas: 8- year OS – 57%; T-lymphoblastic lymphomas: 8-year OS – 27%; Precursor-B-cell lymphoblastic lymphomas: 8- year OS – 52%; Rare NHL: 8-year OS – 30%	10-year OS – 74.1% 10-year EFS – 67.1%
Relative survival rates in case of disease relapse: patients of all ages	3-year OS – 24%; 5-year OS – 10%	5-year OS – 19%	42-month PFS – 73.6%	Diffuse large B-cell lymphomas or high-grade B-cell lymphoma/B-cell lymphoma, unclassifiable: 3-year OS – 24%, 3-year PFS – 14%; Follicular Lymphomas: 5-year OS – 34 – 90%	43-month OS – 83%; 43-month PFS – 68%

OS, overall survival; EFS, Event-free survival; PFS, progression-free survival.

The Surveillance, Epidemiology, and End Results (SEER) database, maintained by the National Cancer Institute (NCI) Data presented as of 21.04.2025.

US Cancer Statistics incidence data cover 99% of the US population and are drawn from population-based registries that participate in the CDC#39; National Program of Cancer Registries (NPCR) and/or the Surveillance, Epidemiology, and End Results (SEER) program of the National Cancer Institute. Rates are per 100,000 and are age-adjusted to the 2000 US Std Population (20 age groups - Census P25-1130).

### 4.3 Clinical evaluation of selected targets in multiple myeloma and non‑Hodgkin’s lymphoma

CAR-T cell therapy targeting BCMA is the most studied and successful target in r/r MM patients (79), but relapse remains a major issue in myeloma treatment. Decreased antigen expression or deletion is a major cause of CAR-T cell treatment failure. The best solution is to find novel CAR-T cell therapy targets. Clinical trials are investigating alternate targets such as GPRC5D, CD138, CD38, CD19, and CS1 in r/r MM patients (79).

One promising target is the G-protein coupled receptor, group 5 class C member D (GPRC5D), expressed on malignant plasma cells with limited expression in normal tissue. Several trials have examined the safety, tolerability, and efficacy of GPRC5D-targeted CAR-T treatment in r/r MM patients (ChiCTR2100048888, 33 patients; NCT04555551, 17 patients; NCT05016778, 10 patients; NCT04674813, 17 patients). The overall response rate of patients included in the study ranged from 71% to 100% (in the ChiCTR2100048888, NCT04555551, NCT05016778, and NCT04674813, the objective response rate of patients with MM was 91%, 71%, 100%, and 86%, respectively), while the complete remission rate of patients analyzed in the ChiCTR2100048888, NCT04555551, and NCT05016778 was 64%, 35%, and 60%, respectively (80). Toxicity of the treatment was limited; grade 3 or higher cytokine release syndrome (CRS) was observed only with the use of GPRC5D-targeted CAR-T, MCARH109, in the clinicaltrials.gov study NCT04555551 and was observed in 1 (6%) patient with r/r MM.

Severe immune effector cell-associated neurotoxicity syndrome (ICANS) (grade 3 or higher) was also limited and was observed in 1 (6%) patient receiving GPRC5D-targeted CAR-T therapy in the NCT04555551 trial, as well as in 1 (3%) patient included in the ChiCTR2100048888 study (80). It is important to note that the analyzed studies included patients with disease progression after BCMA-targeted CAR T-cell therapy. Thus, CAR-T cell therapy directed against GPRC5D demonstrates a high safety and efficacy profile, including patients with disease progression following BCMA-targeted CAR-T cell therapy. Therefore, this approach represents a possible alternative treatment option for patients with MM progression following BCMA-targeted CAR-T cell therapy (81–84).

Several early clinical trials are evaluating the safety, tolerability, and efficacy of CS1-targeted CAR-T cells in r/r MM (NCT04499339, NCT04541368, NCT03710421). The CARAMBA-1 study (NCT04499339) is evaluating the safety and efficacy of autologous CS1-targeted CAR-T cells in patients with late-stage MM for whom conventional therapies have been exhausted. NCT03710421 is evaluating the side effects and appropriate dose of CS1-targeted CAR-T cell treatment after chemotherapy in relapsed or refractory CS1-positive MM patients. Study NCT04541368 is determining the safety and efficacy of CS1-targeted CAR-T cell therapy for relapsed MM following BCMA-targeted CAR-T cell therapy. There are few and ongoing studies on the use of CAR-T cells targeting CD138 (syndecan-1), CD38, SLAMF7, and integrin β7 receptors in patients with r/r MM. In addition, representative results from these clinical studies are currently not available. Studies of CAR-T cells targeting CD38 in r/r are rare (NCT03464916, NCT05442580), the results of which have not yet been published. Anti-CD38 studies are rare due to several factors, including marked extratumoral toxicity due to CD38 receptor expression on normal hematopoietic cells, the availability of drugs (daratumomab, isatuximab) targeting this target, and the high risk of disease relapse in r/r MM due to reduced CD38 expression (85, 86).

Several studies have found that some clones of MM cells express CD19 and define a subpopulation of myeloma-like stem cells (87). Subsequent studies using a combination of BCMA-targeted CAR-T cells and CD19-specific CAR-T cells demonstrated the efficacy of CD19-targeted CAR-T cells (88, 89).

Research on the use of bidirectional CAR-T therapy is promising; it is represented by the combined infusion of two CAR-T cell therapies, as well as the use of bispecific or tandem CAR-T cells. In a number of clinical trials, alternative receptors such as CD138, CD38, CD19, GPRC5D, and CS1 have been used in combination with BCMA to develop dual-acting CAR-T cells for the treatment of r/r MM.

The largest study of bispecific BCMA/CD38-targeted CAR-T cells was presented by Heng Mei et al. A total of 23 patients showed an overall response, which was determined in 20 (87%) of the 23 patients, including 12 (52%) achieving a strict complete response, 4 (17%) achieving a very good partial response (VGPR), and 4 (17%) achieving partial remission. The median progression-free survival was 17.2 months. Median duration of response (DOR) and OS (overall survival) were not reached; 1-year DOR and OS rates were 76% and 93%, respectively. CRS occurred in 87% of patients and was mostly grade 1-2 (65%). Neurotoxicity did not develop in the observed patients. BM38 CAR-T cells were detectable in 77.8% of evaluable patients at 9 months and 62.2% at 12 months (90).

A single-arm phase 1/2a clinical trial (NCT04662099) was designed to evaluate the feasibility, safety, and efficacy of bispecific CS1/BCMA-targeted CAR-T cells in patients with r/r MM. A total of 16 patients received CS1/BCMA-targeted CAR-T cell infusion. Overall response rate to therapy was 81% for all 16 treated patients, including 6 (38%) patients having a stringent complete response (CR), 3 (19%) having VGPR, and 4 (25%) patients having a partial response (PR); 3 (19%) patients did not respond to therapy. CRS was observed in 6 (38%) patients; CSR grade 3 was observed in 1 patient. No neurotoxicity was observed in the observed patients (91).

Ying Wang, Jiang Cao et al. provided intermediate results of a phase II study in patients with r/r MM receiving a combination of BCMA-targeted and CD19-targeted CAR-T cells after lymphodepletion. 62 patients were included in the study, of whom 57 (92%) achieved an overall response, including 20 (32%) strict CR, 17 (27%) CR, 12 (19%) VGPR, and 8 (13%) PR. The median progression-free survival was 18.3 months, and the median overall survival was not reached. Subsequently, 26 of 57 (45%) patients developed disease progression or relapse during the follow-up period. CRS was observed in 59 patients (95%), of whom 10% had grade 3 or higher. Neurotoxic events developed in 7 patients (11%), including 3% of patients with grade 3 or higher (89).

Updated results of the phase I, open-label study of BCMA/CD19 dual-targeting, fast CAR-T GC012F for patients with r/r MM, were reported recently by Juan Du, Wei-Jun Fu, and Hua Jiang (92). GC012F is a CAR-T cell therapy that targets the BCMA and CD19 antigens and was developed using the novel FasT CAR-T platform that ensures the CAR-T cell production within 22–36 hours. The clinical trial studies NCT04236011 and NCT04182581 (clinicaltrials.gov) included 29 patients with r/r MM. After a course of lymphodepletion, patients received one infusion of GC012F. Efficacy assessment demonstrated a high ORR of 93.1% (27/29), with an CR of 82.8% and VGPR of 89.7% (26/29). All patients who received therapy (29/29) scored negative for MRD test. The median DOR was 37.0 months, and the median progression-free survival (PFS) was 38.0 months, respectively. No serious toxicity of the therapy was observed: grade 3 CRS was registered in 2 (6.9%) patients. ICANS was not observed among the patients included in the study (92).

The presented data indicate that to date the most promising alternative target for the treatment of patients with r/r MM is CAR-T directed against GPRC5D (**Table 2**). The results of several studies using GPRC5D-targeted CAR-T cells demonstrate an acceptable safety profile (in most cases, the toxicity of CAR-T therapy was mild to moderate and manageable). Notably, its efficacy was comparable to the standard BCMA-targeted CAR-T, even in patients with relapsed MM after BCMA-specific CAR-T therapy. The overall and complete response rates in patients with r/r MM according to the KarMMa study on the use of Idecabtagene vicleucel, were 73% and 33%, respectively; in the CARTITUDE-1 study on Ciltacabtagene Autoleucel, the analyzed rates were 97% and 67%; and in the ChiCTR2100048888 study on GPRC5D-targeted CAR-T, the overall and complete response rates were 91% and 64%. However, a more detailed comparative evaluation of BCMA- and GPRC5D-targeted CAR-T therapies requires a larger number of treated patients as well as a longer follow-up period. One of the most promising alternative approaches to CAR-T therapy in r/r MM is the use of dual CAR-T cells, which in several studies have demonstrated an impressive efficacy and high safety. In the study on the use of BCMA/CD19 dual-targeting fast CAR-T GC012F the overall and complete response rates were 93.1% and 82.8%, respectively.

**Table 2:** Outcomes of clinical trials evaluating CAR‑T therapies targeting alternative antigens in multiple myeloma.

Author	Agent	Target	Clinical trial ID	Pts (n)	ORR (%)	≥ CR (%)	Grade ≥ 3 CRS (%)	Grade ≥ 3 ICANS (%)
Jieyun Xia et al. (80)	GPRC5D CAR-T	GPRC5D	ChiCTR2100048888	33	91	64	0	3
Sham Mailankody et al. (81)	MCARH109	GPRC5D	NCT04555551	17	71	35	6	6
Mingming Zhang et al. (82)	OriCAR-017	GPRC5D	NCT05016778	10	100	60	0	0
Susan Bal et al. (84)	BMS-986393	GPRC5D	NCT04674813	17	86	–	0	0
Heng Mei et al. (90)	BM38 CAR-Ts	BCMA/CD38	ChiCTR1800018143	23	87	52	17	0
Chenggong Li et al. (91)	CS1+BCMA bispecific CART	CS1-BCMA	NCT04662099	16	81	38	6	0
Ying Wang, Jiang Cao et al (89)	anti-BCMA CAR-T cells and anti-CD19 CAR-T	BCMA/CD19	ChiCTR-OIC-17011272	62	92	59	10	3
Juan Du, Wei-Jun Fu et al (92)	GC012F	BCMA/CD19 dual-CAR-T	NCT04236011; NCT04182581	29	93.1	82.8	6.9	0

The present data indicate that despite a significant number of novel CAR-T therapy approaches in the treatment of patients with r/r MM, relapse remains a major therapeutic challenge. Therefore, further investigation of possible therapeutic options is required.

CD19-targeted CAR-T cell treatment is proven effective in r/r B-cell highly aggressive lymphomas. Intermediate results of the greatest clinical studies of CD19-specific CAR-T cell treatment in highly malignant aggressive B-cell lymphoma (ZUMA-1, JULIET, TRANSCEND) show excellent response and controlled safety.

CD19 loss causes many patients to relapse after CD19-targeted CAR-T treatment, even though most respond. Due to its toxicity, CAR-T cell treatment requires sophisticated, expensive concurrent therapies. Thus, CD22, CD20, BAFFR, CD70, CD5, CD7, TRBC1, and other novel targets for r/r NHL treatment are being intensively studied.

Clinical trials NCT04088890 and ChiCTR1800019298 showed safety and efficacy data for CD22-targeted CAR-T cells in patients with r/r high-risk large B-cell lymphoma (LBCL) and diffuse large B-cell lymphoma (DLBCL) after failure of CD19-targeted CAR-T therapy. In NCT04088890 were enrolled 21 patients. The overall response rate was 86% (determined in 18 patients). Complete remission was determined in 11 (61%) patients; a PR was found in 7 (39%) patients. CRS was observed in all patients; 1/21 (5%) had grade 3 CRS. ICANS was observed in 4 patients (19%); all cases had grade 1–2 severity (93).

Haibo Zhu, Haobin Deng et al. reported the initial results of the ChiCTR1800019298 study, which included 13 patients with r/r diffuse B-cell lymphoma (7 patients) and B-cell ALL (6 patients). As a result of CD22-targeted CAR-T cell therapy, 4 (57%) patients with r/r diffuse B-cell lymphoma achieved complete remission, 2 (28.7%) patients showed PR, and 1 (14.3%) patient had disease stabilization. The OSR of patients with r/r diffuse B-cell lymphoma and patients with r/r ALL who did not receive allogeneic hematopoietic stem-cell transplantation after the CD22-targeted CAR-T cell therapy was 67.07% and 20.5% at 180 days after CAR-T cell therapy, respectively. Progression-free survival of patients with diffuse B-cell lymphoma and patients with r/r ALL are 66.7% and 20.0%. 1–2 grade CRS was diagnosed in 9 of 13 patients, and no patients were diagnosed with ICANS (94).

A noteworthy prospective single-center phase I study, ChiCTR2000036350, demonstrated high efficacy and moderate toxicity of CAR-T cells targeting the CD20 receptor in patients with r/r B-cell NHL treated with rituximab. A total of 15 patients were included in the study. The overall response rate was 100%: 12 (80%) achieved complete remission, and three (20%) achieved partial remission. The median follow-up time was 12.4 months. Progression-free survival and overall survival had not yet been achieved by the end of data collection. No patient developed grade 4 CRS (95).

There are a number of ongoing clinical trials of CAR-T cell therapy targeting BAFFR receptors in patients with r/r B-cell NHL (NCT05370430) and CD70 in patients with CD70-positive malignant hematological diseases, including patients with NHL (NCT04662294). Results of current studies using CD70-targeted CAR-T in patients with lymphoproliferative disorders have not yet been published.

On the related note, Budde, Del Real, et al. reported the initial results of the NCT05370430 study, evaluating the safety and efficacy of autologous BAFFR-targeted CAR-T cells in three patients with B-cell lymphoma (2 patients with mantle cell lymphoma, 1 patient with T-cell/histiocyte-rich B-cell lymphoma). All three treated patients responded to the treatment. The overall response rate was 100%, including minimal residual disease (MRG), negative complete response in two patients and PR in one patient. All three patients developed grade 1 CRS; and two patients developed grade 1 ICANS (96). Wang, Fang, et al. reported the efficacy and safety of tandem CAR-T cells targeting CD19 and CD22, demonstrating a good safety profile of tandem CAR-T cell therapy in patients with r/r NHL. A total of eleven adult patients with r/r NHL (diffuse large B-cell lymphoma, follicular lymphoma, mantle cell lymphoma) were enrolled in the study. Most patients achieved a complete response, demonstrating the efficacy and safety of tandem CD19/CD20 CAR-T cells. Of all enrolled patients, one patient died from infectious complications before evaluation, and ten patients were available for the efficacy evaluation. The overall response rate was 90%; complete remission was determined in seven (70%) patients, and PR was found in two (20%) patients. The median duration of response for the nine responders was 11.83 months. Five patients (45%) developed CRS; among them, one patient (9%) had grade 3 CRS. Two patients (18%) developed ICANS and were graded as grade 3 (97).

Peripheral T-cell lymphoma is one of the most unfavorable histological types of lymphoma. The prognosis for patients with peripheral T-cell lymphoma is unfavorable. The applied therapy is ineffective. With standard anthracycline-based therapy, the complete response rate ranges from 40% to 60%, with an OS of 30-40% (98).

Therefore, it is necessary to search for new approaches to the therapy of this pathology, one of which is CAR-T cell therapy targeting the TRBC1 receptor. The ongoing phase 1/2 study NCT03590574 evaluated the toxicity and efficacy of CAR-T cells targeting the TRBC1 receptor in patients with peripheral T-cell lymphoma. The study included 13 TRBC1-positive patients. A total of ten patients received a single infusion of CAR-T cells after a course of lymphodepletion. One patient who received CAR-T cells achieved complete metabolic response by PET-CT after bridging therapy, hence the response was evaluated in only nine out of ten patients. The best overall response rate (CR and PR) was 66.6% (6 of 9 patients). Complete metabolic response was observed in 4 of 6 responding patients, and 2 patients achieved PR. CRS was observed in 4 of 10 (40%) patients, 1 (10%) patient developed grade 3 CRS, and all CRS events were observed when the maximum dose level was applied. No neurotoxicity was observed in any of the patients, and no dose-limiting toxicity (DLT) was also observed (99).

T-lymphoblastic leukemia/lymphoma (T-ALL/LBL) is a highly aggressive disease. Because the biological, molecular-genetic, and immunophenotypic spectra of alterations in T-lymphoblastic leukemia and T-lymphoblastic lymphoma are comparable, most cooperative groups combine patients with T-ALL and T-lymphoblastic leukemia in a common study (100). Despite the use of intensive chemotherapeutic regimens combined with allogeneic hematopoietic stem cell transplantation, the main failure of therapy for T-cell lymphoproliferative diseases is frequent relapses of the disease. Patients with refractory/relapse T-lymphoblastic leukemia/lymphoma have a poor prognosis. The overall 5-year survival rate of patients with relapsed T-ALL applying standard chemotherapy was 7% (101).

The development of CAR-T therapies for T-cell lymphoma/leukemia is a challenging task complicated by the presence of identical cell markers expressed on the surface of both CAR-T and tumor T-cells. Targeting such T-cell markers by CAR-Ts can lead to the onset of an undesirable effect known as fratricide, in which CAR-T cells destroy each other due to the presence of target epitopes on their surface. Consequently, this process compromises the efficacy of such CAR-T cells in anti-cancer therapy. In addition to the fratricide phenomenon, the presence of malignant T-cells in preparations of autologous CAR-T cells that subsequently undergo the *in vitro* propagation step severely jeopardizes the success of CAR-T-based therapy of T-cell lymphoma/leukemia. These limitations need to be taken into account when developing such therapies against T-cell-associated malignancies.

Currently, the most widely studied target in the treatment of patients with r/r T-lymphoblastic lymphoma/leukemia is the CD7 molecule. In several studies on the use of T-cell receptor-targeted CAR-T cells in patients with r/r T-lymphoblastic lymphoma/leukemia (NCT04004637, NCT04689659, NCT04572308), the use of different methods of blocking the CD7 receptor on both autologous and allogeneic CAR-T cells resulted in high efficacy (overall response rate in patients with r/r T lymphoma/leukemia was as high as 90%) and moderate to severe toxicity (102–104).

The open-label phase I clinical trial NCT04004637 enrolled patients with r/r CD7-positive T-cell acute lymphoblastic leukemia/lymphoma who underwent transfusion of autologous CD7-targeted CAR-T cells. The frequency of complete remission 3 months after CAR-T infusion was 87.5% (in 7 out of 8 patients). Most patients (87.5%) had grade 1 or 2 CRS without T-cell hypoplasia or any neurological toxicity. In this study, an intracellular retention approach was used to block CD7 in CAR-T cells using a tandem CD7 nanobody fused with the endoplasmic reticulum/Golgi-retention motif peptide (102).

High efficacy of CAR-T cell therapy was demonstrated in the NCT04572308 study in patients with r/r T-cell acute lymphoblastic leukemia/lymphoblastic lymphoma. The phase 1 study NCT04572308 enrolled 20 patients with r/r T-cell acute lymphoblastic leukemia (14 patients) and T-cell lymphoblastic lymphoma (6 patients) who received infusion of CAR-T cells targeting the CD7 receptor (NS7CAR). Therapy resulted in 19 (95%) patients achieving complete remission by day 28 of therapy, and 5 of 9 patients achieved extramedullary complete remission. At a median follow-up of 142.5 days after NS7CAR infusion, 14 patients subsequently received allogeneic hematopoietic stem cell transplantation; all patients had no disease relapse. Of the 6 patients who did not receive allogeneic hematopoietic stem cell transplantation, 4 (67%) patients remained in complete remission for an average of 54 days. CRS was detected in 18 (90%) patients, 1 patient (5%) had grade 3 CRS, and 2 had grade 1 neurotoxicity. In this study, no additional manipulations were used to reduce CD7 expression in T-cells, but «naturally selected» CD7-targeted CAR T cells were obtained, in which antigenic masking of CD7 occurred due to the interaction with CD7-specific CAR on the surface of CAR-T cells (104).

In research NCT04689659, r/r T-ALL patients received a single infusion of CD7-specific CAR-T cells from HLA-matched or haploidentical donors who had previously given stem cells to the patients after hematopoietic stem cell transplantation or from new donors. New donors donate stem cells for transplantation to treat bone marrow aplasia and prevent graft-versus-host responses. A total of 20 patients participated. Standard lymphodepletion was conducted in 12 (60%) patients before a single CAR-T cell infusion, while enhanced lymphodepletion was performed in 8 (40%) patients who had not previously undergone transplantation to prevent donor-derived CAR-T cell engraftment failure. A 90% efficacy evaluation response rate (19 individuals). 18 (90%), including 85% (17 patients), achieved MRD-negative complete response by day 15 of therapy. Remission patients had a median follow-up of 6.3 months. After allogeneic hematopoietic stem cell transplantation, 7 (38.9%) patients reached remission, 6 of whom stayed in remission. Nine of the 11 (61.1%) patients who did not receive follow-up therapy were in remission at 7.0 months, while one experienced a CD7-negative recurrence. CRS occurred in all 20 patients (100%) and was grade 3 in 2 (10%). Early grade 1 neurotoxicity occurred in three (15%) individuals. Twelve (60%) patients exhibited early graft-versus-host disease (GVHD), 11 with grade 1 cutaneous and 1 with grade 2 hepatic. In this work, the IntraBlock^®^ technology developed by Shanghai YaKe Biotechnology Ltd. was used to prevent fratricide, which also involves intracellular retention of CD7 by using CD7-binding scFv linked to the endoplasmic reticulum retention signal sequence (103).

Of particular interest is the phase 1 study NCT05032599, which evaluated the efficacy and safety of donor CAR-T cells targeting the CD5 receptor in patients with r/r T-ALL developed after therapy with CAR-T cells targeting the CD7 receptor. In this study, CRISPR/Cas9-based CD5 gene knockout was used to overcome the fratricide effect. Five patients with CD7-negative relapse after CD7-targeted CAR-T therapy were included in the study. After the lymphodepletion stage, patients with previous stem cell transplantation received CAR-T cells from previous hematopoietic stem cell donors, whereas patients who did not receive allogeneic hematopoietic cell transplantation received CAR-T cells from new donors. All 5 patients included in the study achieved complete remission at day 30 and remained MRD-negative with a mean follow-up time of 2.7 months. CRS of grade 1–2 detected in 4 (80%) patients, cutaneous form of graft-versus-host reaction of grade 1–2 was revealed in 4 patients (105).

The studies described above demonstrate the possibility of effective use, and an acceptable safety profile of CAR-T cell therapies directed against several alternative targets such as CD22, CD20, BAFFR, CD5, CD7, and TRBC1 in patients with malignant lymphoproliferative diseases (**Table 3**). In the study presented by Frank MJ, Baird JH et al. on the use of CD22-specific CAR-T therapy in 21 patients with high-risk r/r LBCL and DLBCL after CD19-targeted CAR-T therapy failure, high rates of both overall objective response and complete remission were demonstrated, which were 86% and 61%, respectively (93). These indicators were comparable with those in patients with r/r aggressive B-cell lymphomas receiving CD19-targeted CAR-T therapy in such large studies as ZUMA-1 (n=101, ORR=82% CR=54%) and JULIET (n=115, ORR=53% CR=39%). However, for a more accurate comparison of the presented data, a comparable number of patients and observation periods are required (106, 107). The safety profiles of the CD22-specific CAR-T therapy were satisfactory, since patients experienced mild to moderate severity of CSR and ICANS. Thus, the results of the NCT04088890 and ChiCTR1800019298 studies indicate the possibility of effective therapy for patients with r/r aggressive B-cell lymphomas after CD19-specific CAR-T therapy. One of the prospects of CAR-T cell therapy is the use of tandem CAR-T cells. In the study by Wang L. et al. (97) the efficacy and safety of tandem CD19/20 CAR-T cells were demonstrated in 11 patients with r/r NHL; the ORR and CR rates were 90% and 70%, respectively, while the median duration of response for the 9 responders was 11.83 months (97). In addition, severe CRS and ICANS were observed in isolated cases. Of particular interest is the study reported by Cheng Q, Tan J, and Liu R (95), confirming the effective use of CAR-T cells targeting the CD20 in patients with r/r B-cell NHL treated with rituximab. After the application of CAR-T cells targeting the CD20, 80% of patients achieved CR. The median follow-up time was 12.4 months; in the analyzed group of patients, no serious toxicity (CRS, ICANS) was observed (95). Thus, the application of CD20-specific CAR-T cells was more effective than the use of anti-CD20 monoclonal antibodies.

**Table 3:** Outcomes of clinical trials evaluating CAR‑T therapies targeting alternative antigens in non‑Hodgkin’s lymphoma and leukemia.

Author	Diseases	Target	Clinical trial ID	Pts, n	ORR, %	≥ CR, %	Grade ≥ 3 CRS, %	Grade ≥ 3 ICANS, %
Frank MJ, Baird JH et al. (93)	r/r high-risk LBCL and diffuse large B-celllymphoma (DLBCL) after failure of CD19-directed CAR-T therapy	CD22	NCT04088890 ChiCTR1800019298	21	86	61	5	0
Haibo Zhu, Haobin Deng et al. (94)	r/r diffuse B-cell lymphoma (7 pts) and B-cell ALL (6 pts)	CD22	ChiCTR1800019298	13	67.07 (NHL), 20.5 (ALL)	57 (NHL)	0	0
Cheng Q, Tan J, Liu R et al. (95)	r/r B-cell NHL	CD20	ChiCTR2000036350	15	100	80	0	0
Elizabeth L. Budde, Marissa Morales Del Real et al. (96)	r/r B-cell lymphoma	BAFF	NCT05370430	3	100	66	0	0
Lixin Wang, Chuling Fang et al. (97)	r/r B-cell NHL	CD19/CD20	NCT04723914	11	90	70	9	18
Cwynarski K, Iacoboni G, et al. (99)	Peripheral T-cell lymphoma	TRBC1	NCT03590574	10	66.6	44.4	10	0
Mingzhi Zhang, Dan Chen et al. (102)	r/r T-lymphoblastic lymphoma/leukemia	CD7	NCT04004637	8	–	87.5	–	–
Lu P, Liu Y, Yang J, et al. (104)	r/r T-lymphoblastic lymphoma (6 pts)/leukemia (14 pts)	CD7	NCT04572308	20	–	95	5	0
Pan J, Tan Y, Wang G, et al. (103)	r/r T-cell leukemia	CD7	NCT04689659	20	90	90	10	0
Pan J, Tan Y, Shan L (105).	r/r T-cell leukemia	CD5	NCT05032599	5	–	100	0	0

As mentioned earlier, one of the most serious problems in hematology is T-lymphoblastic malignant lymphoproliferative diseases, since despite the use of intensive chemotherapeutic regimens in combination with allogeneic hematopoietic stem cell transplantation, the main failure of T-cell lymphoproliferative disease therapy is frequent relapses of the disease. The development of CAR-T therapy for T-cell lymphoproliferative diseases is a complex task and faces several limitations, including the identity of cellular markers expressed on the surface of both CAR-T and tumor T-cells. Targeting T-cell markers of CAR-T cells results in a side effect known as fratricide, in which CAR-T cells destroy each other due to the presence of target epitopes on their surface. In addition, there is a risk of expansion of malignant T cells that are present in autologous CAR-T cell preparations. Despite serious limitations in the production of CAR-T cells directed to T-cell markers, several studies (NCT04004637, NCT04572308, NCT04689659) have demonstrated the efficacy and safety of CAR-T cell therapy. Of particular note is a study on the application of CAR-T cells directed to the T-cell marker CD5 in 5 patients with r/r T-ALL that relapsed after therapy with CAR-T cells targeting the CD7 receptor. The study included five patients with CD7-negative relapse after CD7-specific CAR-T therapy. After CD5-targeted CAR-T therapy, all five patients included in the study achieved complete remission. This study demonstrates the possibility of an additional treatment option for patients who have already received CAR-T therapy directed at alternative targets in malignant T-cell lymphoproliferative disease. In sum, the results of these trials demonstrate that CAR-T cell therapy induces a high response rate in patients with r/r lymphomas. Although studies on new targets are still in the early stages, the efficacy of these therapies has already shown encouraging results.

### 4.4 Limitations of the study

Our study may be subject to a certain degree of bias in predicting novel targets for CAR-T therapy. This limitation arises from our reliance primarily on publicly available bulk RNA-Seq data rather than single-cell analysis. As a result, we were unable to precisely assess the extent of immune cell infiltration in the target tissues, which may have influenced the accuracy of our conclusions.

Regarding the integration of RNA and protein expression data, another limitation of our study arises from potential discrepancies between transcriptional and protein expression levels of putative CAR-T targets. Once again, the currently available open-access datasets hinder their direct integration into the analytical pipeline due to differences in sensitivity, spatial resolution, and methodological bias. In our analysis, RNA expression was assessed using consensus RNA expression data from the Human Protein Atlas (RNA-Seq data), while protein expression was derived from immunohistochemistry data based on tissue microarrays from the same source.

Taken together, these limitations reduce the reliability of a strictly quantitative scoring approach. Therefore, we opted for a more illustrative strategy, focusing on the rationale behind target prioritization rather than producing a formal ranking.

## 5 Conclusion

The results of the presented studies demonstrate that CAR-T cell therapy induces a high response rate in patients with hematological malignancies. However, despite the responses to therapy yielding impressive results, relapses of the disease remain a serious problem. Therefore, new targets for CAR-T therapy are being actively investigated, and several studies of CAR-T cell therapy directed at alternative targets have been achieved. Based on our analysis, the most likely targets for clinical approval in disease groups such as lymphoma and multiple myeloma are CD20, CD22 (or in combination with CD19), CD38, BAFFR, CD7, and CD5. Although the studies are early, the efficacy of these therapies has demonstrated encouraging results. These new targets will therefore provide an alternative for patients in relapse following CAR-T therapy applied to common well-known targets in patients with malignant hematological diseases (**Table 4**).

**Table 4:** Pharmaceutical companies developing CAR‑T therapies against emerging antigens: products and clinical trial phases.

Company	Name	Target	Disease	Clinical trail ID	Phase
PeproMene Bio Inc.	PMB-101	BAFFR	r/r B-ALL	NCT04690595	2
	PMB-102		r/r NHL	NCT05370430	2
Cellular Biomedicine Group, Inc.	No data available	CD20	r/r NHL	–	1
Fortress Bio	MB-106	CD20	r/r B-сell NHL, CLL	NCT05360238 NCT03277729	3
Janssen Research & Development, LLC	JNJ-90009530	CD20	B-NHL	NCT05784441	2
Umoja Biopharma	UB-VV300	CD20	Hematological malignancies	–	1
Xyphos Inc.	No data available	CD20	No data available	–	1
Cellectis	UCART22	CD22	r/r B-ALL	NCT04150497	2
Curocell	CRC02	CD22	Lymphoma	–	1
The Pregene (ShenZhen) Biotechnology Company, Ltd.	No data available	CD22	B-ALL	–	1
Sorrento Therapeutics	STI-1492	CD38	r/r MM	NCT05007418	2
	anti-CD38 CAR-T	CD38	r/r MM	NCT03464916	2
Yake Biotechnology Ltd.	CD38 CAR T-cells	CD38	CD38^+^ hematological malignancies	NCT05239689	2
IASO BIO	RD125	CD5	PTCL, MCL, CLL	–	1
The Pregene (ShenZhen) Biotechnology Company, Ltd.	No data available	CD5	T-ALL	–	1
Beam Therapeutics	BEAM-201	CD7	r/r B-ALL, T-ALL, CD7^+^ AML	NCT05885464	3
Hebei Senlang Biotechnology Co., Ltd.	SENL101	CD7	T-ALL, LBL	–	1
Medisix Therapeutics Pte Ltd.	PCART7	CD7	T-ALL, T-LL, AML	–	2
			T-cell malignancies	–	1
Nanjing Bioheng Biotech Co., Ltd.	RD13	CD7	T-ALL	–	1
PersonGen BioTherapeutics (Suzhou) Co., Ltd.	PA3-17	CD7	T-ALL, LBL	NCT05170568	2
Wugen	WU-CART-007	CD7	r/r T-ALL, lymphoblastic lymphoma	NCT04984356	3
Yake Biotechnology Ltd.	CD7 CAR-T cells	CD7	CD7^+^ hematologic diseases	NCT05827835, NCT04599556	3
			T-lymphoid malignancies	NCT04823091	2

AML, acute myeloid leukemia; B-ALL, B-cell acute lymphoblastic leukemia; CLL, chronic lymphocytic leukemia; MM, multiple myeloma; MCL, mantle cell lymphoma; NHL, non-Hodgkin’s lymphoma; PTCL, peripheral T-cell lymphoma; LBL, lymphoblastic lymphoma; T-ALL, T-cell acute lymphoblastic leukemia; TCL, T-cell lymphoma; T-LL, T-lymphoblastic leukemia/lymphoma.
