# Supplementary material for: Future perspectives on novel CAR-T therapeutics beyond CD19 and BCMA in onco-hematology
Source: Front Immunol. 2025 Jul 14;16:1592377. doi: 10.3389/fimmu.2025.1592377 (PMC12301417; doi:10.3389/fimmu.2025.1592377)
Supplement: Supplementary file 1 [file Table1.docx]

Supplementary Material

# Methodology

**Biological characterization of targets**

***CAR-T potential targets***

TRBC1 (ENSG00000211751); CD7 (ENSG00000173762); GPRC5D (ENSG00000111291); BCMA (ENSG00000048462); CD22 (ENSG00000012124); CLL1 (ENSG00000172322); CD70 (ENSG00000125726); CD19 (ENSG00000177455); ROR1 (ENSG00000185483); CD123 (ENSG00000185291); CD5 (ENSG00000110448); CD30 (ENSG00000120949); CS1 (ENSG00000026751); CD33 (ENSG00000105383); BAFFR (ENSG00000159958); CD20 (ENSG00000156738); CD38 (ENSG00000004468).

***Cellular localization of potential target molecules***

Information about the cellular localization of target molecules was obtained from the UniProt database.

***Specificity of potential target genes expression***

Tau specificity score (τ) for normal tissues were analyzed from the Human Protein Atlas and GTEx databases(54). The tau index is commonly used to assess the specificity and breadth of gene expression. Genes which have tau score closer to 1 are expressed more specifically and narrowly in one tissue, genes with tau score close to 0 are expressed ubiquitously and broadly in the tissues under study. There are intervals of Tau specificity score characteristic of a particular expression specificity: ubiquitous or broad gene expression is with τ ≤ 0.5; intermediate gene expression is with 0.5 < τ < 0.9; tissue-specific or narrow gene expression is with τ ≥ 0.9(54).

***Normal tissue RNA and protein expression***

The levels of selected gene RNA expression were analyzed based on RNA consensus tissue gene data from the Human Protein Atlas (HPA), available via <https://v23.proteinatlas.org./download/rna_tissue_consensus.tsv.zip> (Human Protein Atlas version 23.0 and Ensembl version 109). There wasn’t any information about RNA expression in amygdala, basal ganglia, cerebellum, hippocampal formation, hypothalamus, midbrain, pituitary gland, spinal cord and vagina for TRBC1 (ENSG00000211751) gene.

The «Normal tissue groups» were categorized based on HPA RNA consensus tissue gene data: «Adipose tissue» (adipose tissue), «Muscle» (skeletal muscle, tongue, smooth muscle), «Heart muscle» (heart muscle), «Gastrointestinal» (colon, duodenum, esophagus, gallbladder, rectum, small intestine, stomach), «Nervous» (amygdala, basal ganglia, cerebellum, cerebral cortex, choroid plexus, hippocampal formation, hypothalamus, midbrain, pituitary gland, spinal cord), «Immune system» (appendix, bone marrow, lymph node, spleen, thymus, tonsil), «Skin» (skin), «Endocrine system» (adrenal gland, parathyroid gland, thyroid gland), «Salivary gland» (salivary gland), «Lungs» (lung), «Urinary system» (kidney, urinary bladder), «Liver» (liver), «Pancreas» (pancreas), «Eye» (retina), «Male tissue» (epididymis, prostate, seminal vesicle, testis), «Female tissue» (breast, cervix, endometrium, fallopian tube, ovary, placenta, vagina).

In order to clarify the level of RNA expression of selected genes in immune cells, data from «RNA HPA immune cell gene data» archive available by link <https://v23.proteinatlas.org./download/rna_immune_cell.tsv.zip> were processed (Human Protein Atlas version 23.0 and Ensembl version 109). To compare the RNA expression levels in tissue groups and immune cells normalized expression (nTPM) data was used, where TPM is transcripts per million.

Immune cells were grouped based on «RNA HPA immune cell gene data»: «basophil» (basophil), «eosinophil» (eosinophil), «neutrophil» (neutrophil), «monocytes» (intermediate monocyte, classical monocyte, non-classical monocyte), «naive B-cell» (naive B-cell), «memory B-cell» (memory B-cell), «naive CD4 T-cell» (naive CD4 T-cell), «naive CD8 T-cell» (naive CD8 T-cell), «memory CD4 T-cell» (memory CD4 T-cell), «memory CD8 T-cell» (memory CD8 T-cell), «MAIT T-cell» (MAIT T-cell), «γδ T-cell» (γδ T-cell), «T-reg» (T-reg), «NK-cell» (NK-cell), «Dendritic cells» (plasmacytoid DC, myeloid DC) and «total PBMC» (total PBMC).

Expression profiles for proteins of selected genes were analyzed based on the HPA archive available from <https://v23.proteinatlas.org./download/normal_tissue.tsv.zip> (Human Protein Atlas version 23.0 and Ensembl version 109). Groups of tissues for protein expression were same as for RNA expression, except that no data were available for protein expression in following organs: tongue, amygdala, basal ganglia, choroid plexus, hippocampal formation, hypothalamus, midbrain, pituitary gland, spinal cord, thymus and retina. Protein expression in the hippocampus was added into the group «Nervous». Also, HPA database doesn’t include information about protein expression profiles of following genes: TRBC1 (ENSG00000211751), BCMA (ENSG00000048462), CD70 (ENSG00000125726), ROR1 (ENSG00000185483).

Tissue consensus RNA expression of gene in normal tissue groups were defined as «high» if the mean log_2_(nTPM+1) of the tissue group was from 8.9 to 5.94, «medium» – from 5.94 to 2.98 and «low» – from 2.98 to 0.00. Tissue consensus RNA expression of genes in immune cells were defined as «high» if the mean log_2_(nTPM+1) of the tissue group was from 11.7 to 7.8, «medium» – from 7.8 to 3.9 and «low» – from 3.9 to 0.0. The calculation involved counting down each third (representing «high», «medium», and «low» expression) from the maximum level of the mean value of log_2_(nTPM+1). Protein expression of each gene was defined as «high - medium» and «low - not detected» based on the information from the HPA database. If the tissue contains different cells with both «high – medium» and «low – not detected», the expression was classified as «variable». If there wasn’t any information about protein expression, it was classified as «unknown».

# Table S1. 24 selected publicly available targets (data from pharmaceutical companies’ websites, Autumn 2023).

| **Targets** | **Pharmaceutical companies** | **Drug name** | **Hematological malignancy** | **Clinical trial ID** |
| --- | --- | --- | --- | --- |
| **Single targeting (approved antigenes)** | | | | |
| CD19 | AbClon Inc | AT101 | B-NHL | NCT05338931 |
|  | Allogene | ALLO-501A + ALLO-647 (anti-CD52 mAb) | r/r LBCL | NCT05714345 |
|  | Allogene | ALLO-501A | r/r LBCL | NCT04416984 |
|  | Atara Biotherapeutics | ATA3219 | B-cell malignancies | - |
|  | Atara Biotherapeutics | ATA3219 | B-cell malignancies | - |
|  | Autolus Limited | Obecabtagene autoleucel | B-ALL | NCT04404660 |
|  | Autolus Limited | Obecabtagene autoleucel | B-NHL, CLL | NCT02935257 |
|  | Autolus Limited | Obecabtagene autoleucel | Primary CNS lymphoma | NCT04443829 |
|  | Autolus Therapeutics | Obecabtagene autoleucel | r/r B-ALL | NCT04404660 |
|  | Avalon Globocare, Corp. | AVA-001 | B-ALL, NHL | NCT03952923 |
|  | Caribou Biosciences, Inc. | CB-010 | r/r B-NHL | NCT04637763 |
|  | Caribou Biosciences, Inc. | CB-010 | r/r B-NHL | NCT04637763 |
|  | Celularity, Inc. | CYCART-19 | NHL, MCL | - |
|  | Celularity, Inc. | CYCART-201 | NHL, MCL | - |
|  | Chongqing Precision Biotech | pCAR-19B | B-ALL | NCT05334823 |
|  | CRISPR Therapeutics | CTX112 | B-cell malignancies | NCT05643742 |
|  | CRISPR Therapeutics | CTX110 | r/r B-cell malignancies | NCT04035434 |
|  | Curocell | CRC01 | Large B-cell lymphoma | NCT04836507 |
|  | Fate Therapeutics | FT819 | r/r B-cell lymphoma, r/r CLL, and r/r precursor B-ALL | NCT04629729 |
|  | Gracell Biotechnologies | GC007g | B-ALL | - |
|  | Gracell Biotechnologies | GC019F | B-ALL | NCT04595162 |
|  | Gracell Biotechnologies | GC007g | B-ALL | - |
|  | Hebei Senlang Biotechnology Co., Ltd. | Senl 1904B | B-ALL | - |
|  | Hrain Biotechnology | CD19 targeted DASH CAR-T Cells | B-ALL | NCT05651191 |
|  | Hrain Biotechnology | CD19 CAR-T | r/r B-NHL | NCT05436223 |
|  | Hrain Biotechnology | Anti-CD19 CAR-T | B-ALL | NCT03798509 |
|  | Hrain Biotechnology | CD19 CAR-T | DLBCL, FL | NCT03720457 |
|  | Immuneel Therapeutics | Varnimcabtagene autoleucel | B-cell malignancies | CTRI/2022/03/041162 IMAGINE Study |
|  | Immunochina Pharmaceuticals | IM19 | DLBCL | NCT04440436 |
|  | Immunochina Pharmaceuticals | IM19 | B-ALL | NCT05309213 |
|  | Immunochina Pharmaceuticals | IM19 | MCL | NCT05155215 |
|  | Juventas Cell Therapy Ltd. | Inaticabtagene Autoleucel | ALL | NCT04684147, NCT05667506 |
|  | Juventas Cell Therapy Ltd. | HY001（CNCT19) Inaticabtagene Autoleucel | NHL | NCT04586478 |
|  | JW Therapeutics | Relmacabtagene Autoleucel (Carteyva) | 3L LBCL | marked |
|  | JW Therapeutics | Relmacabtagene Autoleucel (Carteyva) | 3L FL | marked |
|  | JW Therapeutics | Relmacabtagene Autoleucel (Carteyva) | 3L MCL | - |
|  | JW Therapeutics | Relmacabtagene Autoleucel (Carteyva) | Front line LBCL | - |
|  | JW Therapeutics | Relmacabtagene Autoleucel (Carteyva) | 2L LBCL | - |
|  | JW Therapeutics | Relmacabtagene Autoleucel (Carteyva) | 3L ALL | - |
|  | JW Therapeutics | Relmacabtagene Autoleucel (Carteyva) | 3L CLL | - |
|  | Kecellitics Biotech Company Ltd | No data available | B-cell leukemia | NCT04100187 |
|  | Kite Pharma, Inc. | Axicabtagene ciloleucel (Yescarta) | NHL | NCT02348216 (ZUMA-1) |
|  | Kite Pharma, Inc. | Axicabtagene ciloleucel (Yescarta) | 1L high-risk DLBCL | NCT05605899 (ZUMA-23) |
|  | Kite Pharma, Inc. | Axicabtagene ciloleucel (Yescarta) | 2L high-risk FL | NCT05371093 (ZUMA-22) |
|  | Kite Pharma, Inc. | Axicabtagene ciloleucel (Yescarta) | 2L DLBCL outpatient | NCT05459571 (ZUMA-24) |
|  | Kite Pharma, Inc. | Brexucabtagene autoleucel (Tecartus) | Pediatric B-ALL, NHL | NCT02625480 (ZUMA-4) |
|  | Kite Pharma, Inc. | Axicabtagene ciloleucel (Yescarta) | DLBCL | NCT03391466 (ZUMA-7) |
|  | Kite Pharma, Inc. | Brexucabtagene autoleucel (Tecartus) | Rare B-cell malignancies | NCT05537766 (ZUMA-25) |
|  | Nektar Therapeutics | NKTR-255 + Yescarta or Breyanzi | DLBCL | NCT05664217 |
|  | Nektar Therapeutics | NKTR-255 + Breyanzi | r/r DLBCL | NCT05359211 |
|  | Novartis | YTB323 (Rapcabtagene autoleucel) | Adult B-ALL | NCT03960840 |
|  | Novartis | Tisagenlecleucel (Kymriah) | B-ALL | NCT02435849 (ELIANA) |
|  | Novartis | YTB323 (Rapcabtagene autoleucel) | 1L high-risk DLBCL | NCT03960840 |
|  | Precision BioSciences | azer-cel; PBCAR0191 | r/r B-ALL, r/r B-NHL | NCT03666000 |
|  | Precision BioSciences | PBCAR19B | r/r NHL | NCT04649112 |
|  | Servier | S95023/S68587 | B-ALL | NCT02808442 |
|  | Shanghai GeneChem Co., Ltd. | GB5005 | Leukemia/lymphoma | - |
|  | Takara Bio Inc. | TBI-2001 | r/r CD19^+^ B-cell Lymphoma, CLL, SLL | NCT05963217 |
|  | Takeda Pharmaceutical | TAK-940 | r/r B-cell malignancies | NCT04464200 |
|  | Takeda Pharmaceutical | TAK-940 | r/r B-cell malignancies | NCT04464200 |
|  | Tianjin Mycure Medical Technology Co., Ltd | CD19 CAR-T | B-ALL | NCT03671460 (status:unknown) |
|  | TiCARos, Co., Ltd. | TC011 | lymphoma | - |
|  | TiCARos, Co., Ltd. | TC012 | lymphoma | - |
|  | Timmune Biotech Inc. | TI-1007 | B-NHL | NCT03720496 (status:unknown) |
|  | Timmune Biotech Inc. | CD19-TriCAR-SILK | Leukemia, NHL | NCT03910842 (status:unknown) |
|  | Umoja Biopharma | UB-VV111 | Hematological malignancies | - |
|  | UniCAR Therapy | U-01 | B-ALL | - |
|  | UWELL Biopharma | UWC-19 | MCL | NCT04296461 (status:unknown) |
|  | UWELL Biopharma | UWC-19 | r/r CD19^+^ Leukemia and lymphoma | NCT03811457 |
|  | Verismo Therapeutics | SynKIR-310 | post-CAR r/r DLBCL | - |
|  | Yake Biotechnology Ltd. | CD19 CAR-T | Lymphoma | NCT05239676, NCT04661020 |
|  | Yake Biotechnology Ltd. | Murine CD19 CAR-T | B-ALL, B-NHL | NCT04532281 |
|  | Yake Biotechnology Ltd. | Humanized CD19 CAR-T | B-ALL, B-NHL | NCT04532268 |
| BCMA | Allogene | ALLO-715 | r/r MM | NCT04093596 |
|  | Allogene | ALLO-605 | r/r MM | NCT05000450 |
|  | Arcellx | ACLX-001 | r/r MM | NCT04155749 |
|  | Caribou Biosciences, Inc. | CB-011 | r/r MM | NCT05722418 |
|  | Caribou Biosciences, Inc. | CB-011 | r/r MM | NCT05722418 |
|  | CARsgen Therapeutics | Zevor-cel (CT053, zevorcabtagene autoleucel) | r/r MM | NCT03975907 (LUMMICAR 1) |
|  | CARsgen Therapeutics | Zevor-cel (CT053, zevorcabtagene autoleucel) | r/r MM | NCT03915184 (LUMMICAR 2) |
|  | CARsgen Therapeutics | Zevor-cel (CT053, zevorcabtagene autoleucel) | r/r MM | - |
|  | CARsgen Therapeutics | CT0590 | r/r MM | - |
|  | Cartesian Therapeutics | Descartes-08 | r/r MM | NCT04816526, NCT03448978 |
|  | Cartesian Therapeutics | Descartes-11 | r/r MM | NCT04436029, NCT03994705 |
|  | Cellular Biomedicine Group, Inc. | No data available | r/r MM | - |
|  | Celyad Oncology SA | CYAD-211 | r/r MM, B-NHL | NCT04613557 |
|  | CRISPR Therapeutics | CTX120 | r/r MM | NCT04244656 |
|  | CRISPR Therapeutics | CTX121 | Hematological malignancies | - |
|  | Curocell | CRC03 | r/r MM | - |
|  | Eureka Therapeutics | No data available | r/r MM | - |
|  | Hebei Senlang Biotechnology Co., Ltd. | SENL103 | r/r MM | - |
|  | Hebei Senlang Biotechnology Co., Ltd. | SENL302 | r/r MM | - |
|  | Hrain Biotechnology | BCMA CAR-T | r/r MM | NCT05594797 |
|  | Hrain Biotechnology | Anti-BCMA CAR-T | r/r MM | NCT05302648, NCT04003168 |
|  | IASO BIO | CT103A | r/r MM | NCT05698303 |
|  | IASO BIO | CT103A | high-risk ND MM | - |
|  | IASO BIO | CT103A | r/r MM | NCT05066646 FUMANBA-1 |
|  | Immunochina Pharmaceuticals | IM21 | r/r MM | NCT05478343 |
|  | Immunochina Pharmaceuticals | INS21(InstanCART) | r/r MM | - |
|  | Janssen Research & Development, LLC (Johnson & Johnson) | Ciltacabtagene Autoleucel | r/r MM | NCT04923893 (CARTITUDE-5) |
|  | Janssen Research & Development, LLC (Johnson & Johnson) | CAR- PRISM | Smoldering myeloma | NCT05767359 |
|  | Janssen Research & Development, LLC (Johnson & Johnson) | Ciltacabtagene Autoleucel | r/r MM | NCT05257083 (CARTITUDE-6) |
|  | JW Therapeutics | JWCAR129 | r/r MM | - |
|  | Kite Pharma, Inc. | CAR-T ddBCMA (Kite-772) (iMMagine-1) | r/r MM | NCT05396885 |
|  | Legend Biotech | LCAR-BCDR | r/r MM | NCT05376345 |
|  | Legend Biotech | LUCAR-B68 | r/r MM | NCT05498545 |
|  | Legend Biotech | LCAR-BCDR Cells | r/r MM | NCT05376345 |
|  | Legend Biotech | LCAR-B38M Cells | r/r MM | NCT03090659 (LEGEND-2) |
|  | Legend Biotech | LCAR-B38M CAR-T Cell | r/r MM | NCT03758417 (CARTIFAN-1) |
|  | Legend Biotech | JNJ-68284528 (Ciltacabtagene Autoleucel) | r/r MM | NCT03548207 (CARTITUDE-1) |
|  | Legend Biotech | JNJ-68284528 (Ciltacabtagene Autoleucel) | r/r MM | NCT04133636 (CARTITUDE-2) |
|  | Legend Biotech | JNJ-68284528 (Ciltacabtagene Autoleucel) | r/r MM | NCT04181827 (CARTITUDE-4) |
|  | Legend Biotech | JNJ-68284528 (Ciltacabtagene Autoleucel) | ND MM | NCT04923893 (CARTITUDE-5) |
|  | Legend Biotech | JNJ-68284528 (Ciltacabtagene Autoleucel) | ND MM | NCT05257083 (CARTITUDE-6) |
|  | Poseida Therapeutics | P-BCMA-ALLO1 | r/r MM | NCT04960579 |
|  | Protheragen Inc. | ***0004 | r/r MM | - |
|  | The Pregene (ShenZhen) Biotechnology Company, Ltd. | PRG-1801 | MM, PLC | NCT03661554 |
|  | UniCAR Therapy | U-51 | r/r MM | - |
|  | Yake Biotechnology Ltd. | BCMA CAR-T | r/r MM | NCT05740891, NCT05430945, NCT04670055 |
|  | Yake Biotechnology Ltd. | BCMA CAR-T | r/r MM | NCT05712083 |
| **Dual targeting** | | | | |
| CD19/CD22 | Autolus Limited | AUTO1/22 | Pediatric B-ALL | NCT02443831 |
|  | Century Therapeutics | CNTY-102 | B-cell lymphoma, B-cell malignancies | - |
|  | Eureka Therapeutics | No data available | B-cell lymphoma, B-cell lymphoma | - |
|  | Exuma Biotech | rPOC-752 | B-cell lymphoma | - |
|  | Hrain Biotechnology | CD19/CD22 targeted T-cells | r/r B-cell malignancies | NCT05651178, NCT05223686 |
|  | IASO BIO | CT120 | B-NHL | NCT05091541 |
|  | IASO BIO | CT120 | B-ALL | - |
|  | Juventas Cell Therapy Ltd. | HY004 | B-ALL, B-NHL | NCT06009107, NCT06005649 |
|  | Kecellitics Biotech Company Ltd | No data available | B-cell lymphoma | NCT05651100 |
|  | Miltenyi Biotec GmbH | MB-CART 2219.1 | Pediatric B-ALL | - |
|  | Nanjing Bioheng Biotech Co., Ltd. | CTA101 | B-ALL, NHL | - |
|  | Nanjing Bioheng Biotech Co., Ltd. | СТА301 | B-ALL, NHL | - |
|  | Nektar Therapeutics | NKTR-255 + CD19/22.BB.z-CAR-T | B-ALL | NCT03233854 |
|  | Protheragen Inc. | Bi-CART | r/r ALL | - |
|  | UniCAR Therapy | CD19/CD22 CAR-T | MCL | - |
|  | Yake Biotechnology Ltd. | CD19/CD22 CAR T | B-ALL | NCT04788472, NCT04740203 |
| CD19/CD20 | Cellular Biomedicine Group, Inc. | No data available | r/r NHL | - |
|  | Janssen Research & Development, LLC (Johnson & Johnson) | JNJ-90014496 | B-NHL | NCT05421663 |
|  | Kite Pharma, Inc. | CD19/20 bicistronic (KITE-363) | 3L^+^ DLBCL | NCT04989803 |
|  | Miltenyi Biotec GmbH | Zamtocabtagene autoleucel | 3L DLBCL | NCT04792489 |
|  | Miltenyi Biotec GmbH | Zamtocabtagene autoleucel | 2L DLBCL | NCT04844866 |
|  | Miltenyi Biotec GmbH | Zamtocabtagene autoleucel | 1L DLBCL | - |
|  | Miltenyi Biotec GmbH | Zamtocabtagene autoleucel | B-cell lymphoma | - |
|  | Miltenyi Biotec GmbH | Zamtocabtagene autoleucel | MCL | - |
|  | Poseida Therapeutics | P-BCMACD19 - ALLO1 | B-cell malignancies | - |
|  | Poseida Therapeutics | P-BCMACD19 - ALLO1 | B-cell malignancies | - |
| BCMA/CD19 | Autolus Limited | AUTO8 | r/r MM | NCT04795882 |
|  | Autolus Therapeutics | AUTO8 | r/r MM | NCT04795882 |
|  | Autolus Therapeutics | AUTO8 | r/r MM | NCT04795882 |
|  | Gracell Biotechnologies | GC012F | ND MM | NCT04236011 |
|  | Gracell Biotechnologies | GC012F | B-NHL | - |
|  | Gracell Biotechnologies | GC012F | r/r MM | NCT05412329 |
|  | Poseida Therapeutics | P-BCMACD19 - ALLO1 | r/r MM | - |
| CD20/CD22 | Cellectis | UCART20x22 | r/r B-NHL | NCT05607420 |
|  | Yake Biotechnology Ltd. | CD20/CD22 CAR-T | Lymphoid Hematological Malignancies | NCT04283006 |
| BCMA/CD7 | Gracell Biotechnologies | GC508 | r/r MM | - |
| CD19/CD7 | Gracell Biotechnologies | GC502 | B-ALL | - |
| CD33/CLL1 | Prescient Therapeutics | No data available | AML | - |
|  | Legend Biotech | LCAR-AMDR | AML | NCT05654779 |
| **Multiple targeting** | | | | |
| CD19/CD20/CD22 | Legend Biotech | LCAR-AIO | ALL, NHL | NCT05318963, NCT05292898 |
| **Other therapeutic targets** | | | | |
| CD20 | Cellular Biomedicine Group, Inc. | No data available | r/r NHL | - |
|  | Fortress Bio | MB-106 | r/r B-cell NHL, r/r B-cell CLL | NCT05360238, NCT03277729 |
|  | Janssen Research & Development, LLC (Johnson & Johnson) | JNJ-90009530 | B-NHL | NCT05784441 |
|  | Umoja Biopharma | UB-VV300 | Hematological malignancies | - |
|  | Xyphos Inc. | Not disclosed | Undisclosed | - |
| CD22 | Cellectis | UCART22 | r/r B-ALL | NCT04150497 |
|  | Curocell | CRC02 | Lymphoma | - |
|  | The Pregene (ShenZhen) Biotechnology Company, Ltd. | No data available | B-ALL | - |
| CD33 | 2seventy bio | SC-DARIC33 | r/r AML | NCT05105152 |
|  | 2seventy bio | SC-DARIC33 | r/r AML | NCT05105152 |
|  | Precigen | PRNG-3006 | r/r AML, MDS | NCT03927261 |
|  | Vor Biopharma | VCAR33 | r/r AML | NCT03971799 |
|  | Vor Biopharma | VOR33/VCAR33 | AML | NCT04849910 |
| CD123 | Arcellx | ACLX-002 | r/r MM, MDS | - |
|  | Cellectis | UCART123 | r/r AML | NCT03190278 |
|  | Hebei Senlang Biotechnology Co., Ltd. | SENL401 | AML | - |
|  | Immunochina Pharmaceuticals | IM23 | AML | NCT03585517 |
|  | Mustang Bio | MB-102 | r/r AML, BPDCN | NCT02159495, NCT04109482 |
|  | Yake Biotechnology Ltd. | IL3 CAR-T | CD123^+^ AML | NCT04599543 |
| CD30 | Tessa Therapeutics Ltd | TT11X | r/r HL, r/r NHL | NCT04288726 |
|  | Tessa Therapeutics Ltd | TT11 | r/r HL | NCT04268706 |
|  | Tessa Therapeutics Ltd | TT11 | HL | NCT05352828 |
| CD38 | Sorrento Therapeutics | STI-1492 | r/r MM | NCT05007418 |
|  | Sorrento Therapeutics | Anti-CD38 CAR-T | r/r MM | NCT03464916 |
|  | Yake Biotechnology Ltd. | CD38 CAR-T | CD38^+^ Hematological Malignancies | NCT05239689 |
| GPRC5D | Bristol Myers Squibb | BMS-986393; CC-95266 | r/r MM | NCT04674813 |
|  | CARsgen Therapeutics | CT071 | r/r MM | - |
|  | Eureka Therapeutics | MCARH109 | r/r MM | NCT04555551 |
| **Novel antigens** | | | | |
| CD70 | CRISPR Therapeutics | CTX130 | Hematological malignancies | NCT04502446 |
|  | TCR2 Therapeutics | TC-520 | Hematological malignancies | - |
|  | Yake Biotechnology Ltd. | CD70 CAR-T | CD70^+^ Hematological malignancies | NCT04662294 |
| CLL1 | Kite Pharma, Inc. | CLL-1 (KITE-222) | r/r AML | NCT04789408 |
|  | Yake Biotechnology Ltd. | CLL1 CAR-T | CLL1^+^ hematological malignancies | NCT05252572 |
|  | Caribou Biosciences, Inc. | CB-012 | r/r AML | - |
| CS1 | Cellectis | UCARTCS1A | r/r MM | NCT04142619 |
|  | T-CURX | SLAMF7 CAR-T | r/r MM | NCT04499339 |
|  | Yake Biotechnology Ltd. | CS1 CAR-T | r/r MM | NCT04541368 |
| BAFFR | PeproMene Bio Inc. | PMB-101 | r/r B-ALL | NCT04690595 |
|  | PeproMene Bio Inc. | PMB-102 | r/r NHL | NCT05370430 |
| CD5 | IASO BIO | RD125 | PTCL, MCL, CLL | - |
|  | The Pregene (ShenZhen) Biotechnology Company, Ltd. | No data available | T-ALL | - |
| ROR1 | Precigen | PRNG-3007 | CLL, MCL, ALL, DLBCL | NCT05694364 |
| TRBC1 | Autolus Limited | AUTO4 | TRBC1+ peripheral TCL | NCT03590574 |

first-line, 1L; second-line, 2L; third-line, 3L; acute myeloid leukemia, AML; B-cell acute lymphoblastic leukemia, B-ALL; blastic plasmacytoid dendritic cell neoplasm, BPDCN; chronic lymphocytic leukemia, CLL; central nervous system leukemia, CNSL; diffuse large B-cell lymphoma, DLBCL; follicular lymphoma, FL; Hodgkin lymphoma, HL; large B-cell lymphoma, LBCL; mantle cell lymphoma, MCL; myelodysplastic syndromes, MDS; multiple myeloma, MM; newly diagnosed, ND; non-Hodgkin's lymphoma, NHL; PLC; peripheral T-cell lymphoma, PTCL; small lymphocytic lymphoma , SLL; T-cell acute lymphoblastic leukemia, T-ALL; T-cell lymphoma, TCL
